# Non-native Douglas fir seedlings outcompete native Norway spruce, silver fir and Scots pine under dry and nutrient-poor conditions

**DOI:** 10.3389/fpls.2025.1546250

**Published:** 2025-03-20

**Authors:** Barbara Moser, Esther R. Frei, Christoph Bachofen, Thomas Wohlgemuth, Daniel Scherrer

**Affiliations:** ^1^ Plant Regeneration Ecology, Forest Resources and Management Unit, Swiss Federal Institute for Forest, Snow and Landscape Research WSL, Birmensdorf, Switzerland; ^2^ Alpine Environment and Natural Hazards, Mountain Ecosystems Unit, WSL Institute for Snow and Avalanche Research SLF, Davos, Switzerland; ^3^ Plant Ecology Research Laboratory PERL, School of Architecture, Civil and Environmental Engineering, EPFL, Lausanne, Switzerland; ^4^ Functional Plant Ecology, Community Ecology Unit, Swiss Federal Institute for Forest, Snow and Landscape WSL, Lausanne, Switzerland; ^5^ Forest Dynamics Unit, Swiss Federal Institute for Forest, Snow and Landscape Research WSL, Birmensdorf, Switzerland

**Keywords:** climate change, common garden experiment, drought tolerance, European beech, nonnative species, nutrients

## Abstract

Climate change is expected to significantly alter forest ecosystems, reducing the suitability of the key economic tree species Norway spruce (*Picea abies*) and European beech (*Fagus sylvatica*) in low- and mid-elevation forests of Central Europe. As these species face increasing pressures from drought, storms, and pests, it is crucial to identify alternative tree species that are economically viable and capable of maintaining primary ecosystem services. This study investigated the potential of Douglas fir (*Pseudotsuga menziesii*), a non-native conifer, to establish from seed and compete with native broadleaf and conifer species during the early regeneration stage under differing resource availabilities. We assessed the growth performance and phenotypic plasticity of Douglas fir seedlings over three years in a controlled common-garden experiment. Seedlings of Douglas fir, along with seven native species — Norway spruce, silver fir (*Abies alba*), Scots pine (*Pinus sylvestris*), European beech, pedunculate oak (*Quercus robur*), sessile oak (*Q. petraea*), and sycamore (*Acer pseudoplatanus*) — were grown for three years under factorial combinations of high and low availabilities of light, nutrients, and water. Seedling height, biomass allocation to shoots and roots and phenotypic plasticity of these traits were measured to evaluate the competitive ability of individual species and their potential to adapt to changing environmental conditions. While Douglas fir seedlings exhibited strong growth performance compared to the conifers Norway spruce and silver fir, their biomass production and height growth was considerably lower than that of the broadleaved sycamore and beech. However, Douglas fir’s height growth rate in the third year exceeded all species except sycamore. This was particularly pronounced under dry and/or nutrient-poor conditions, indicating a potential competitive advantage under expected future climatic conditions. In agreement with field studies, our results indicate that non-native Douglas fir may sustainably establish in dry, nutrient poor European lowland forests due to its superior early growth performance under these conditions and the high phenotypic plasticity, of its root system. This holds especially in situations where the species competes with other conifers, while its ability to successfully compete with broadleaves appears to be largely restricted to nutrient-poor sites.

## 1 Introduction

Climate warming, combined with increasing frequencies of extreme events and disturbances (Seidl et al., 2014, 2017a), will alter species distributions (Lenoir et al., 2008; Dyderski et al., 2018) and shift the dominance of individual species in forest communities (Fensham et al., 2015; Scherrer et al., 2022). Currently, over 60% of the standing wood biomass in Swiss forests is comprised of just two species: Norway spruce (43%; *Picea abies* (L.) H. Karst.) and European beech (18%; *Fagus sylvatica* L.; Cioldi et al., 2020). For both species, the area with suitable growing conditions is expected to decline in the future, especially at low- and mid-elevation (400 – 1000 m a.s.l.), where most timber is currently produced (Gessler et al., 2024). Therefore, potential tree species that could replace these species and maintain the ecosystem services they provide in the long term should be carefully evaluated.

European beech is the naturally dominant broadleaf species at lower elevations in Switzerland (Scherrer et al., 2021), while Norway spruce was actively planted in these areas for timber production (Bürgi and Schuler, 2003). Although outside of its natural distribution range, Norway spruce grows very fast on these sites, which are warmer with respect to the species’ natural range. At the same time, the higher temperatures increase the species’ susceptibility to winter storms (Schütz et al., 2006; Scherrer et al., 2022), drought (Lévesque et al., 2013; Vitasse et al., 2019a), and bark beetle attacks (Obladen et al., 2021; Scherrer et al., 2023). More frequent summer droughts and repeated bark beetle infestations have already started to turn Norway spruce from a bread-and-butter tree to an ultimate loser, not only in the Swiss lowland forests but in many regions of Central Europe (Seidl et al., 2017b; Scherrer et al., 2022). European beech is also getting under increasing pressure, already showing early signs of maladaptation on sites at the drier end of its distribution in Northeastern Switzerland (Schuldt et al., 2020; Frei et al., 2022a). Consequently, alternative tree species are being sought to substitute the economically important Norway spruce in production forests at low elevations and potentially, in the longer term, also European beech at drier sites. From the pool of native species, potential candidates are almost exclusively broadleaf species (Frehner et al., 2019) such as European beech as a substitute for Norway spruce on moist sites and at high elevations, and oaks (*Quercus petraea* Liebl., *Q. robur* L., *Q. pubescens* Willd.), limes (*Tilia cordata* Mill., *T. platyphyllos* Scop.), or maples (*Acer pseudoplatanus* L., *A. campestre* L., *A. platanoides* L.) to replace European beech on drier lowland sites. However, sawmills and related cascade uses of wood products are primarily adapted for coniferous wood, with over 75% of broadleaf wood being used solely for energy production (Swiss Federal Statistical Office, 2024). Consequently, the wood industry would prefer to replace Norway spruce with other conifer species so that the existing processing chains can be maintained (Vor et al., 2015; Pluess et al., 2016).

One of the most promising candidates in Central Europe is the non-native Douglas fir (*Pseudotsuga menziesii* (Mirb.) Franco), which can adapt to a wide range of site conditions, including mesic sites currently stocked with Norway spruce or dry sites populated by oak. Douglas fir is expected to cope well with projected climate conditions in this part of Europe due to its superior drought tolerance compared to native species like Norway spruce and silver fir (*Abies alba* Mill.; e.g., Eilmann and Rigling, 2012; Lévesque et al., 2014; Vitali et al., 2017, 2018; Gazol et al., 2022). Its drought resistance is attributed to several physiological and anatomical adaptations including a higher water use efficiency achieved through effective stomatal regulation, which minimizes water loss while maintaining photosynthetic activity during periods of drought (Smit and van den Driessche, 1992; Jassal et al., 2009), a more resilient xylem structure that reduces the risk of cavitation (Sperry and Ikeda, 1997), as well as osmotic adjustments to maintain cell turgor and metabolic function during drought stress (Kavanagh et al., 1999; Du et al., 2015). These mechanisms collectively contribute to the robust performance and expected resilience of adult trees under the increasingly dry summer conditions anticipated for Central Europe due to climate change. This contrasts with scarce regeneration in the introduced range (Neophytou et al., 2019; Frei et al., 2022b) suggesting that the species has difficulties to establish and to compete with native species during the seedling phase (Moser et al., 2016).

Since its introduction from North America to Europe in the 19th century (Kohnle et al., 2021), Douglas fir has been planted for its high productivity and wood quality (González-García et al., 2013), primarily on productive sites, where its natural regeneration potential is limited and the seedlings are outnumbered by rapidly growing broadleaves such as European beech, ash (*Fraxinus excelsior L.*) or sycamore (Frei et al., 2022b). Only recently has it been shown that adult Douglas fir is more drought tolerant than Norway spruce (Lévesque et al., 2014; Vitali et al., 2017) and might therefore also grow successfully on drier sites. Since plantations with older, seed-producing Douglas firs are still rare on dry sites (Frei et al., 2022b), it remains an open question how competitive Douglas fir performs under drier conditions and different soil fertility levels (Moser et al., 2016; Frei et al., 2022b; Gazol et al., 2022). Evidence from Southern Germany suggests that Douglas fir saplings are able to outcompete native tree species on dry, nutrient poor sites (Knoerzer and Reif, 1996; Essl, 2005; Bindewald and Michiels, 2018), which could be linked to the extensive root system of Douglas fir seedlings in the upper soil layers (Moser et al., 2016) where most of the nutrients are located.

Despite the advantages in productivity and wood quality, there are also concerns about ecological impacts of Douglas fir. Pure Douglas fir stands negatively affect the abundance of several species groups (Wohlgemuth et al., 2021) such as fungi (Buée et al., 2011; Schmid et al., 2014), birds (Utschick, 2006), and insects (Gossner and Simon, 2002; Gossner and Utschick, 2004). However, as a potential candidate for future low- and mid-elevation timber production, and provider of other ecosystem services, Douglas fir’s competitive strength under a wide range of environmental conditions needs to be assessed.

We investigated the potential of Douglas fir to fill the gaps left by declining growth performance of Norway spruce and European beech suffering from drought in Central European lowland forests both under current and expected future conditions (Schuldt et al., 2020). For this, we tested the performance (i.e., height growth rate and biomass production) of Douglas fir seedlings growing in competition with native conifer and broadleaf tree seedlings under varying combinations of light, nutrient and water availability during three years after emergence from seeds. In particular, we analyzed how the species’ phenotypic plasticity with respect to tree height and biomass partitioning between shoot and roots affects its competitive ability under varying environmental conditions compared to the conifers Norway spruce, silver fir and Scots pine (*Pinus sylvestris* L.), and the broadleaves European beech, pedunculate oak, sessile oak and sycamore.

## 2 Methods

### 2.1 Study location and species

**Figure 1:**
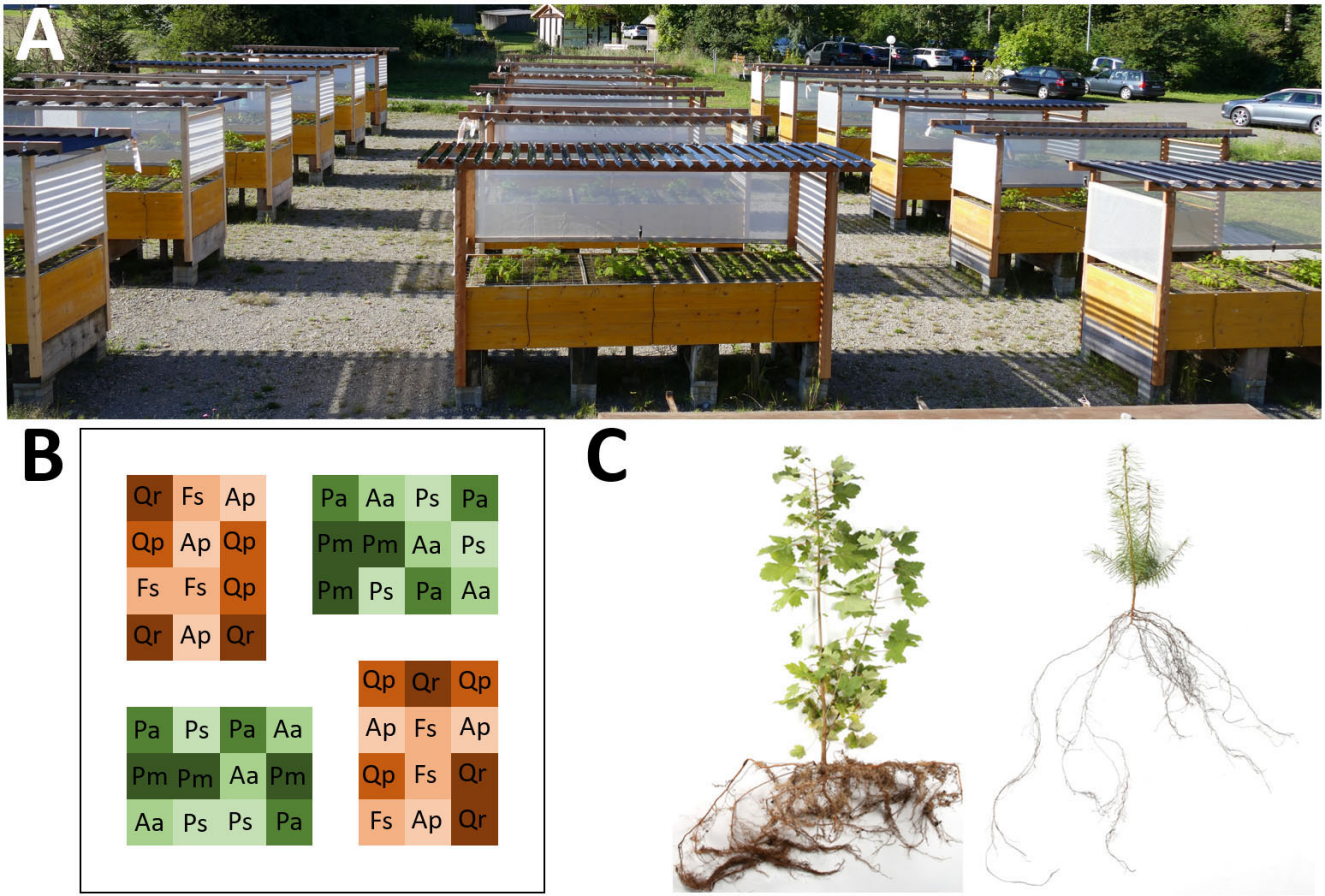
**(A)** Experimental setup with three blocks (rows), each containing all factorial combinations of water availability (ambient precipitation, drought treatment), light availability (low and high shading), and nutrient availability (three levels of fertilization, nested within water × light availability). **(B)** An example single mesocosm (split-plot) separated into 48 squares (split-split plots) each containing one of our eight study species (i.e. six replicates per species per mesocosm). **(C)** Example of a harvested three-year-old sycamore and Douglas fir including their root network.

To simulate natural regeneration in a mixed temperate forest, we sowed seeds of Douglas fir (*Pseudotsuga menziesii* (Mirb.) Franco) and seven principal tree species of temperate Central European lowland forests in mesocosms (1 m × 1 m surface, 0.5 m depth; **Figure 1A**) in a common garden at the Swiss Federal Research Institute WSL (47°21’38.3” N, 8°27’16.6” E, 550 m a.s.l.) in April 2016. The site experiences a temperate climate with a mean annual temperature of 9.3°C and an average annual precipitation of 1134 mm. In addition to our focal species Douglas fir, the experiment included the three native conifers silver fir (*Abies alba* Mill.), Norway spruce (*Picea abies* (L.) H. Karst.), and Scots pine (*Pinus sylvestris* L.) as well as the four native broadleaf species sycamore (*Acer pseudoplatanus* L.), European beech (*Fagus sylvatica* L.), pedunculate oak (*Quercus robur* L.), and sessile oak (*Quercus petraea* Liebl.; seed sources see **Supplementary Table S1**).

### 2.2 Experimental setup

The experimental design was a split-split-plot replicated 3 times (blocks), with precipitation and light as whole-plot factors, nutrients as the split-plot factor (mesocosm), and species as the split-split-plot factor (Zhang et al., 2020). Each mesocosm was divided into 48 sowing quadrats of 10 cm × 10 cm, with 3 – 10 seeds sown per quadrat in April of year 1 (six quadrats per species in each mesocosm; **Figure 1B**). Due to extremely low seedling emergence in European beech, additional seedlings from the same seed lot but grown in the nursery were planted in empty quadrats in September of year 1. The number of seedlings was reduced to equal numbers (three for conifers, two for broadleaves) after leaf-out in spring of year 2, and to one seedling per quadrat in October of year 2 by randomly removing the surplus.

Water availability was manipulated from May to October using throughfall reduction shelters, allowing for either 100% (ambient precipitation) or 50% (drought treatment) of ambient precipitation. To ensure consistent germination and seedling establishment, all mesocosms were regularly watered from April to mid-July and no drought treatment has been applied in year 1. The total precipitation from May to October was 729 mm (ambient) and 607 mm (drought) in year 1, 619 mm and 294 mm in year 2, and 672 mm and 340 mm in year 3. During dry periods, mesocosms were manually watered to match the long-term average May to October precipitation for the region.

Light availability was manipulated from May (leaf out of beech in the neighboring forest) to October (leaf fall) by means of shade cloth with differing light transmittance and different shading of the throughfall reduction channels (**Figure 1A**; for more details see Zhang et al., 2020). In the medium shade treatment, the mesocosms received 38.8 ± 0.021% (mean ± SE) of photosynthetically active radiation (PAR; measured on three days in August and September 2018), which corresponds to a medium size canopy gap of 20 – 25 m. In the light shade treatment, PAR reached 58.0 ± 0.022%, roughly equaling a canopy gap of 35 – 40 m (Gálhidy et al., 2006).

The soil substrate, consisting of 40% quartz sand, 20% fibric peat, 20% expanded schist, 16% pumice, and 4% clay, was composed to have a neutral pH (7.3 CaCl) to accommodate all eight tree species, and low nutrient content (0.0014/<0.001/0.018 g N/P/K m²) to allow nutrient manipulation. Nutrient manipulations were done by adding Gesal Floranid slow-release lawn fertilizer twice a year in April and August. Total nutrient additions were 4.1/1.3/3.7, 8.7/2.9/8.6, and 17.3/5.7/17.1 g N/P/K m² in the low, medium, and high nutrient treatments, respectively, in year 1, and 6/1.5/2.4, 12/3/4.8, and 24.0/6.0/9.6 g N/P/K m² in years 2 and 3. The medium nutrient treatment was designed to provide sufficient nutrients for tree seedling growth, simulating the nutrient levels typically found in mesic beech forests (e.g. *Galio odorati-Fagetum*) across Switzerland (Meier et al., 2005). In contrast, the low nutrient treatment deprives plants of essential nutrients, while the high nutrient treatment offers a luxuriant supply of nutrients. Fertilizer additions were increased in the second and third growing seasons to account for growing plant biomass and nutrient demand.

### 2.3 Measurements after three years

The height of the seedlings was measured at the end of the growing seasons in year 1, 2 and 3, while above ground biomass (dry weight) was assessed after harvest in autumn in year 3. For a random subset of 10 plants per species and treatment combination, the roots were carefully excavated and their biomass measured (**Figure 1C**). To calculate fine root biomass fraction, the roots of one block (4 individuals per treatment combination, only high and low nutrient mesocosms) were separated into fine (< 1mm root diameter) and coarse (> 1mm root diameter) root biomass.

### 2.4 Data analysis

Seedling height and 3^rd^ year height growth increment were considered proxies for the competitive ability of a seedling individual. In forest ecosystems, light is one of the key limited resources and fast height growth is essential for seedlings to compete with surrounding vegetation (Bachofen et al., 2019). The fraction of below ground biomass with respect to total biomass (root biomass fraction) and the fraction of fine roots with respect to total root biomass (fine root biomass fraction) are characteristics of a species’ resource investment strategy. Their variation across treatments, i.e. phenotypic plasticity, shows the degree, to which a species is able to adapt to different environments.

Linear Mixed Effects Models (LMEs) were employed to evaluate the influence of *species* identity (8 levels), *light* (2 levels), *water* (2 levels), and *nutrient* (3 levels), as well as their interactions, on tree seedling height, biomass allocation to shoot (above-ground) and roots (below-ground), root biomass fraction, and fine root biomass fraction. Since all overall models with *species* as fixed factor showed significant *species × environment* interactions, we also run LMEs for individual species. These analyses were conducted using the R package `lme4` (Pinheiro and Bates, 2006; Pinheiro et al., 2023). All models incorporated *mesocosms* nested within *blocks* as random factors to account for the spatial arrangement of the experimental setup (**Figure 1A**). Response variables were log-transformed when necessary to ensure normal distribution and homogeneity of variance. In addition, species-specific phenotypic plasticity was estimated for different traits using the Relative Distance Plasticity Index (RDPI; Valladares et al., 2006), which takes into account the variation of a given trait among treatment combinations.

Our analysis focused on the performance of Douglas fir in comparison to the seven native species, rather than a complete pairwise comparison of all species. To achieve this, we compared for each species the individual plant measurement to the mean value of Douglas fir within the same treatment combination (i.e., log response ratio). We calculated the log response ratio of the seven native species for tree height, above and below ground biomass, root biomass fraction, and fine root biomass fraction for all factorial combinations of *light*, *water*, and *nutrient* availability. To identify significant differences between each native species and Douglas fir within each treatment combination, we used one-sample t-tests, corrected for multiple-testing according to Benjamini and Hochberg (1995).

## 3 Results

### 3.1 Effects of light, water and nutrient availability on tree seedlings

After three years of growth, plant height, 3^rd^ year height growth increment, total biomass, above ground biomass, below ground biomass, and the root biomass fraction, were most and foremost determined by species identity (**Supplementary Table S2**). As a result, variation among species was high, with sycamore outperforming all other species (**Supplementary Figures S1**-**S6**). Treatment effects (i.e., *light*, *water* and *nutrient* availability) were much smaller and differed among species, with significant *species × nutrients* and *species × water* interactions for all traits (**Table 1**; **Supplementary Material**). The addition of fertilizer resulted in a smaller root biomass fraction in all species, i.e. the seedlings allocated less biomass to roots under conditions of medium to high nutrient content compared to low nutrient content (**Supplementary Figure S7**; **Table 1**). Sycamore showed the most consistent reaction to nutrient addition, producing taller shoots along with more above and below ground biomass in the medium and high fertilizer treatments (**Table 1**). Similar to sycamore, Scots pine and pedunculate oak grew taller in the high fertilizer treatment but had comparable biomass in all fertilizer treatments, while silver fir produced more shoot biomass but not taller shoots. By contrast, high nutrient content led to lower height growth increments in the 3^rd^ year in sycamore, Scots pine, and Douglas fir. Water availability mainly affected sessile oak and Scots pine, with high precipitation leading to shorter shoots and, in the case of sessile oak, also lower above and below ground biomass (**Table 1**). In most species, light did neither affect seedling height nor biomass allocation. Only sycamore and beech produced higher root biomass in large canopy gaps (58% light availability), while beech also produced more above ground biomass (**Table 1**).

**Table 1:** The influence of nutrient, water and light availability on diverse plant traits after 3 years of seedling growth based on individual species Linear Mixed Effects models (LMEs).

	Plant height			Height growth			Shoot biomass			Root biomass			Total biomass			Root biomass fraction		
Species	N	W	L	N	W	L	N	W	L	N	W	L	N	W	L	N	W	L
Douglas fir	**-**	**-**	**-**	**↓**	**-**	**-**	**-**	**-**	**-**	**-**	**-**	**-**	**-**	**-**	**-**	**↓**	**-**	**-**
Norway spruce	**-**	**-**	**-**	**-**	**-**	**-**	**-**	**-**	**-**	**-**	**-**	**-**	**-**	**-**	**-**	**↓**	**↑**	**-**
Scots pine	**↑**	**↓**	**-**	**↓**	**-**	**-**	**-**	**-**	**-**	**-**	**-**	**-**	**-**	**-**	**-**	**↓**	**-**	**-**
Silver fir	**-**	**-**	**-**	**-**	**-**	**-**	**↑**	**-**	**-**	**-**	**-**	**-**	**-**	**-**	**-**	**↓**	**-**	**-**
Sycamore	**↑**	**-**	**-**	**↓**	**-**	**-**	**↑**	**-**	**-**	**↑**	**-**	**↑**	**↑**	**-**	**-**	**↓**	**-**	**↑**
European beech	**-**	**-**	**-**	**-**	**-**	**-**	**-**	**-**	**↑**	**-**	**-**	**↑**	**-**	**-**	**↑**	**↓**	**-**	**-**
Sessile oak	**-**	**↓**	**-**	**-**	**-**	**-**	**-**	**↓**	**-**	**-**	**↓**	**-**	**-**	**↓**	**-**	**↓**	**-**	**-**
Pedunculate oak	**↑**	**-**	**-**	**-**	**-**	**-**	**-**	**-**	**-**	**-**	**-**	**-**	**-**	**-**	**-**	**↓**	**-**	**-**

N, Nutrients (low [reference level], medium, high); W, Water (50% of ambient [reference], ambient); L, Light (38% [reference], 50%). All the underlaying LMEs can be found in **Supplementary Material**.

**↑↓** Significant increase or decrease (p < 0.05).

### 3.2 Competitive ability of Douglas fir

After three years of seedling growth, Douglas fir outperformed native silver fir and Norway spruce in total biomass, above ground biomass, below ground biomass, and fine root biomass under limiting conditions—such as low nutrient availability, smaller canopy gaps, and drought treatments (**Supplementary Figures S9**-**S12**). The difference between species was particularly pronounced when multiple stressors were combined. The third native conifer, Scots pine, however, had higher total biomass and shoot biomass than Douglas fir, while root and fine root biomass were similar between the two species (**Supplementary Figures S9**-**S12**). In comparison with broadleaved natives, Douglas fir exhibited lower total biomass, above ground biomass (with the exception of sessile oak), root biomass, and fine root biomass (**Supplementary Figures S9**-**S12**).

**Figure 2:**
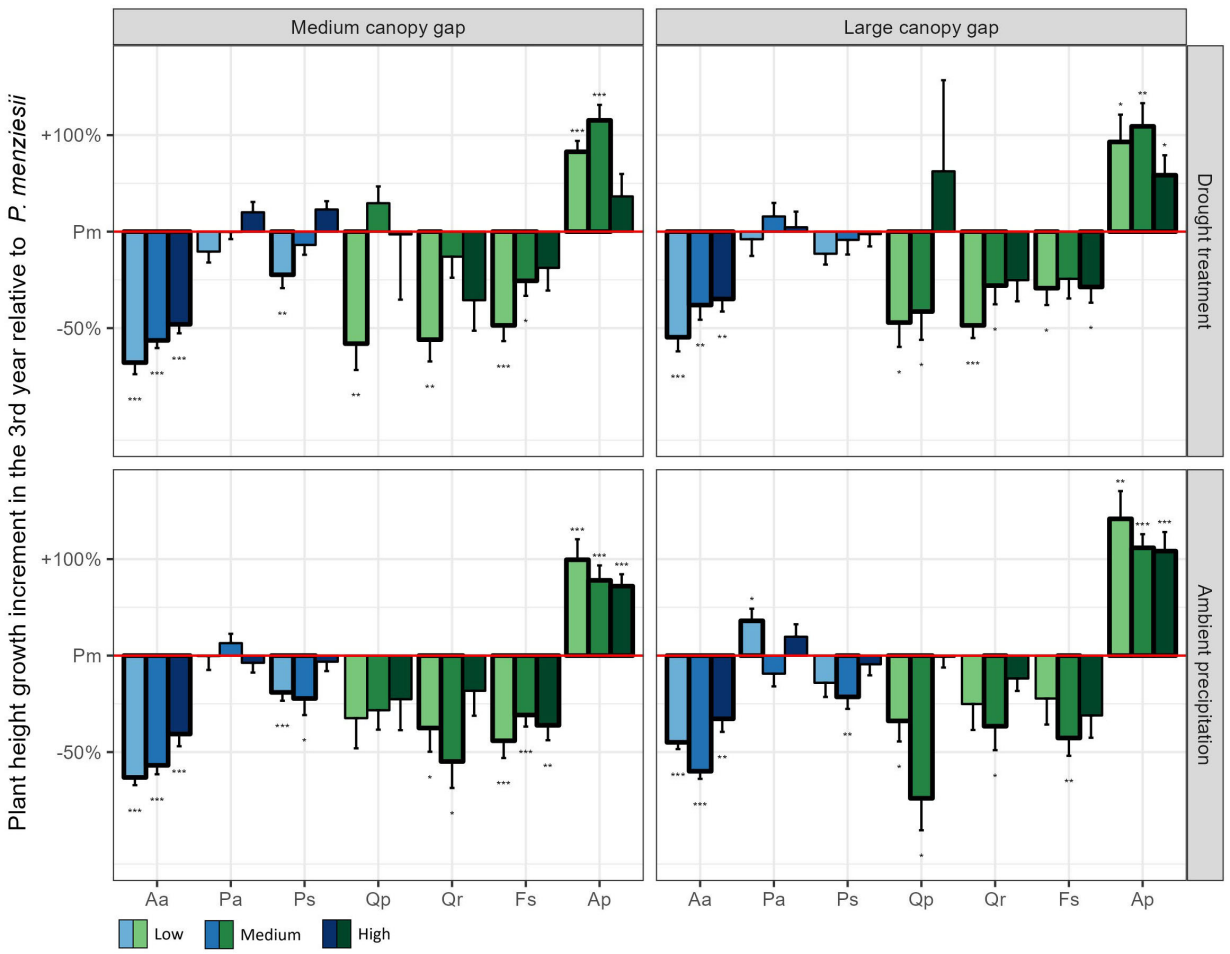
Plant height growth increment in the 3^rd^ year of the experiment for the seven native plant species compared to Douglas fir across all possible light, water, and nutrient treatments. The values of all native plant species were standardized by the corresponding value of Douglas fir in the same treatment combination. Low, medium, and high indicate the three nutrient levels. Aa = Silver fir, Pa = Norway spruce, Ps = Scots pine, Pm *=* Douglas fir, Qp = Sessile oak, Qr = Pedunculate oak, Fs = European beech, Ap = Sycamore. *p < 0.05, **p < 0.01, ***p < 0.001.

**Figure 3:**
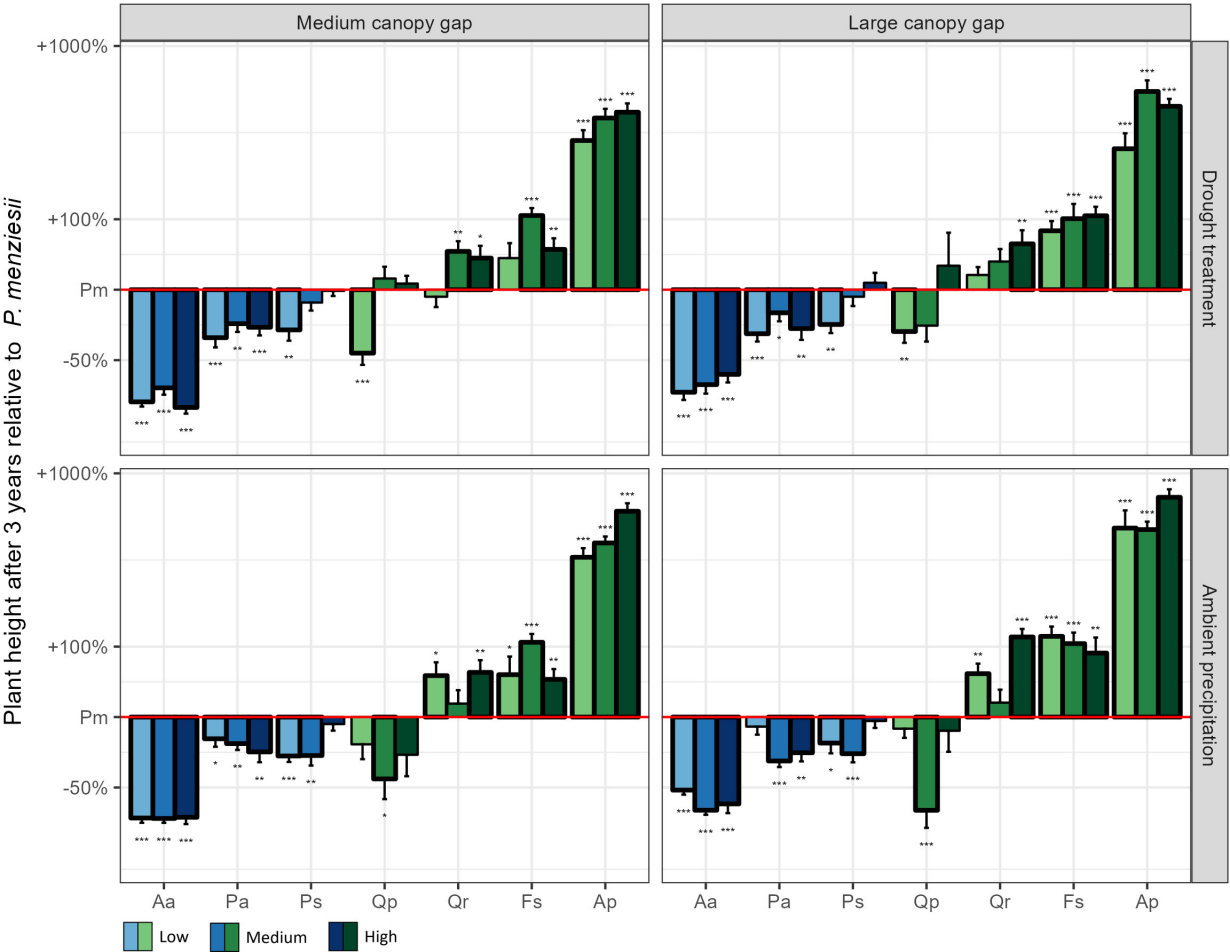
Plant height after three years of the experiment for the seven native plant species compared to Douglas fir across all possible light, water, and nutrient treatments. The values of all native plant species were standardized by the corresponding value of Douglas fir in the same treatment combination. Low, medium, and high indicate the three nutrient levels. Aa = Silver fir, Pa = Norway spruce, Ps = Scots pine, Pm *=* Douglas fir, Qp = Sessile oak, Qr = Pedunculate oak, Fs = European beech, Ap = Sycamore. *p < 0.05, **p < 0.01, ***p < 0.001.

The 3^rd^ year height growth increment of Douglas fir, Norway spruce and Scots pine exceeded that of the oaks and beech in most treatment combinations (**Figure 2**). Notably, Douglas fir had the highest 3^rd^ year height growth increment among these species when all resources were limited (*medium gap* × *drought* × *low nutrients*; **Figure 2**). Consequently, after three years it achieved the highest plant height of all conifers, surpassing even sessile oak (**Figure 3**).

### 3.3 Biomass partitioning and phenotypic plasticity

**Figure 4:**
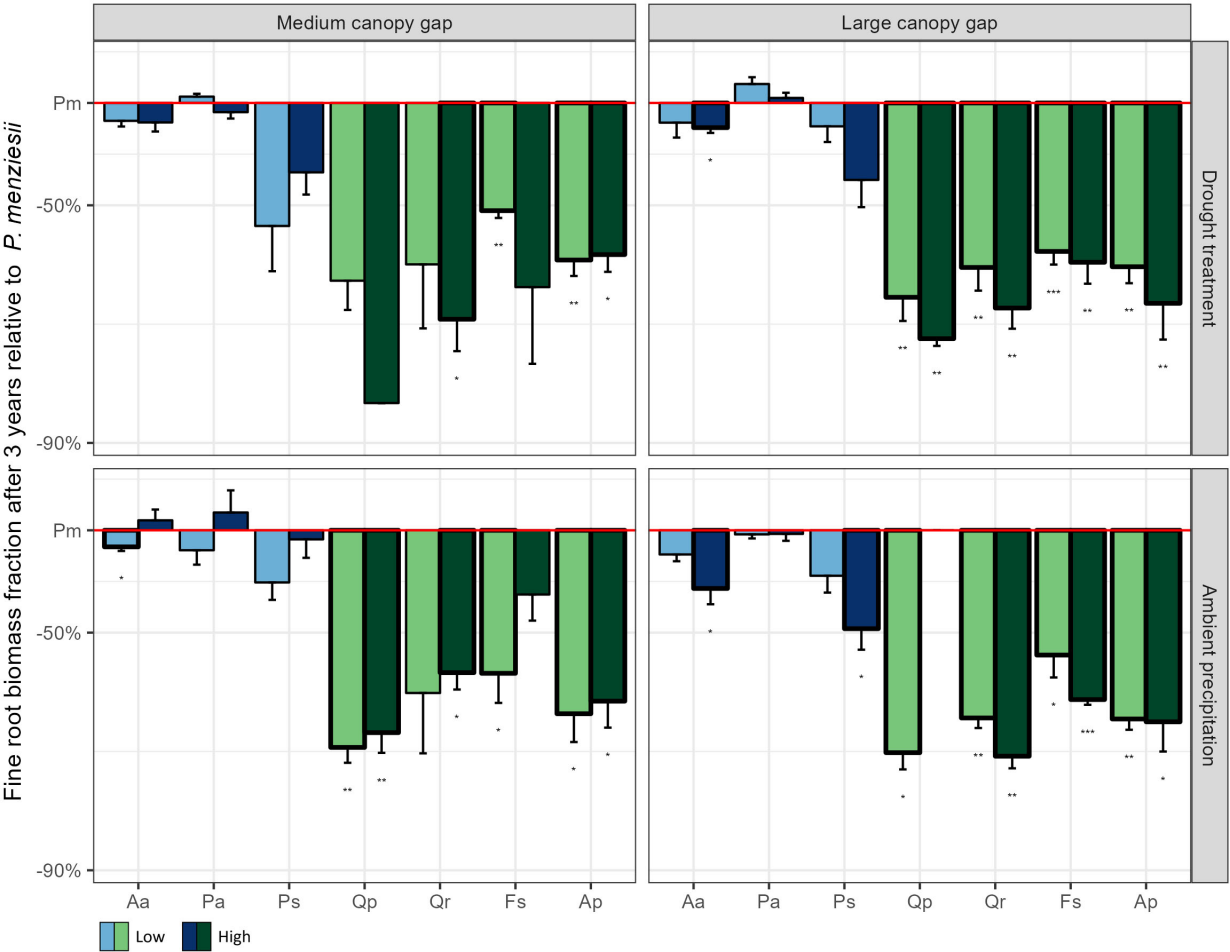
Fine root biomass fraction (fine root biomass/below ground biomass) after three years of the experiment for the seven native plant species compared to Douglas fir across all possible light, water, and nutrient treatments. The values of all native plant species were standardized by the corresponding value of Douglas fir in the same treatment combination. Low, medium, and high indicate the three nutrient levels. Aa = Silver fir, Pa = Norway spruce, Ps = Scots pine, Pm *=* Douglas fir, Qp = Sessile oak, Qr = Pedunculate oak, Fs = European beech, Ap = Sycamore. *p < 0.05, **p < 0.01, ***p < 0.001.

The conifers exhibited substantially higher fine root biomass fractions than broadleaf species (**Figure 4**). While Douglas fir generally had the highest below ground biomass among conifers, it displayed significantly lower below ground biomass compared to broadleaf species (**Supplementary Figure S11**). However, this difference is largely due to broadleaf species developing substantial taproots, whereas Douglas fir primarily invests in fine roots.

**Figure 5:**
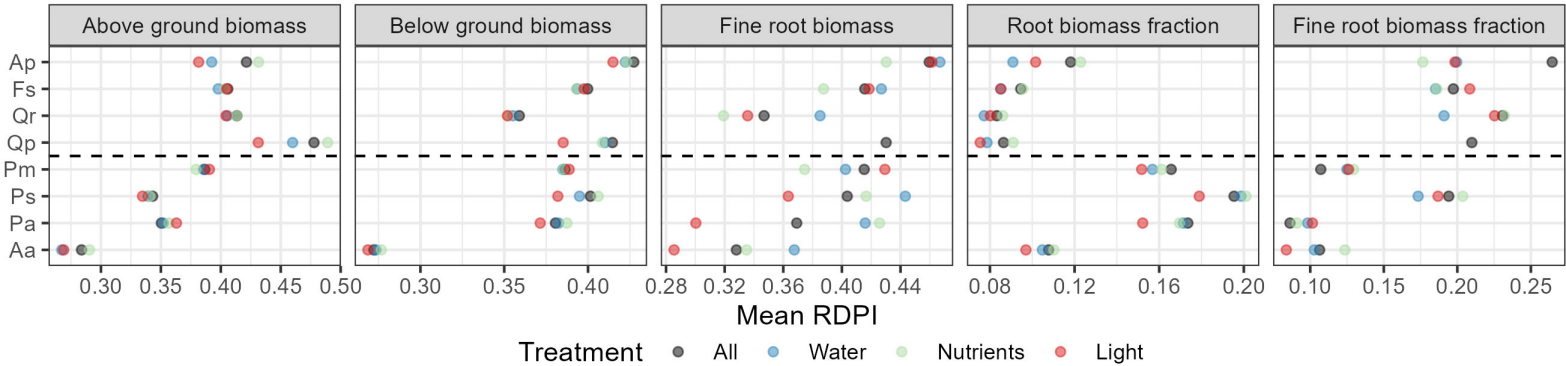
Relative Distance Plasticity Index (RDPI) of the eight different tree species and across the different treatments. All = Across all treatment combinations, Water = Water availability (2 levels), Nutrients = Nutrient availability (3 levels), Light = Light availability (2 levels). Aa = Silver fir, Pa = Norway spruce, Ps = Scots pine, Pm *=* Douglas fir, Qp = Sessile oak, Qr = Pedunculate oak, Fs = European beech, Ap = Sycamore.

Broadleaf species demonstrated greater plasticity in above ground biomass compared to conifers, while variations in below ground biomass and fine roots were similar in both species groups, with the notable exception of silver fir, which exhibited very low plasticity across all traits (**Figure 5**). Conifers, including Douglas fir, adjusted to the environment with a highly plastic root biomass fraction, whereas broadleaves adapted their fine root biomass fraction, instead (**Figure 5**). Nutrient addition triggered the largest phenotypic adaptations in terms of shoot and root biomass, and root biomass fraction, surpassing those induced by drought or light conditions (**Figure 5**). In the conifers, this was also true for fine root biomass, while water availability was the more important driver of fine root biomass allocation in broadleaves.

## 4 Discussion

After its introduction to Europe, Douglas fir was mainly planted on productive sites, so that today’s seed-producing stands only cover a small environmental gradient in Switzerland (Frei et al., 2022b). With our experimental set-up, we aimed to test the competitive ability of Douglas fir compared to key native tree species also on less fertile sites and under different canopy gap sizes. Our results show that the performance of Douglas fir, compared to native tree species, is highly dependent on resource availability, indicating that its ability to regenerate in a competitive environment might differ among sites. While sycamore, beech, and sessile oak consistently grew taller and produced more biomass than Douglas fir across all treatment combinations, Douglas fir consistently outperformed silver fir, which exhibited the smallest stature and lowest biomass. This difference in growth rates is well-documented, as silver fir generally shows very slow growth during its early years. Silver fir compensates for its slow start with exceptional shade tolerance (Niinemets and Valladares, 2006), allowing it to thrive for extended periods under closed canopies (Dobrowolska et al., 2017). This adaptability enables silver fir to eventually reach the canopy even in dense forest stands, as it can persist in the understory longer than many other species. Consequently, silver fir may outperform Douglas fir under very low light conditions, such as those found under a closed canopy — conditions that were not tested in this study. Additionally, long-term observations have revealed that silver fir possesses a high degree of drought resistance and exhibits better recovery and growth after drought compared to Norway spruce (Vitasse et al., 2019b), not being as robust as Douglas fir, though (Vitali et al., 2017).

The competitive outcome between Douglas fir and pedunculate oak, Scots pine and Norway spruce was, in contrast, more complex and differed among traits and resource availability. While pedunculate oak accumulated similar amounts of biomass as Douglas fir in the first three years, Scots pine produced more, and Norway spruce less biomass. In terms of tree height, however, Douglas fir seedlings outcompeted all three species, especially under low nutrient conditions. To gain height quickly is important for tree seedlings in order to escape competition for light from accompanying vegetation and browsing pressure by deer (Bachofen et al., 2019). The exceptional height growth increments of Douglas fir in the 3^rd^ year, which exceeded those of all other species except sycamore, indicate that Douglas fir would catch up in height with beech or sessile oak in the following years, and outperform pedunculate oak, Scots pine, Norway spruce and silver fir even more. The fact that height growth increments of Douglas fir were particularly increased compared to most other tree species under conditions of low resource availability (low nutrients combined with low water and/or lower light) suggests that its competitive ability is particularly pronounced on dry, nutrient poor sites. Even though these findings are based on a common garden experiment, they align very well with several studies in forest ecosystems in Austria and southern Germany, where natural regeneration of Douglas fir was particularly abundant in dry, resource poor oak forests (Knoerzer and Reif, 1996; Essl, 2005; Bindewald and Michiels, 2018). These results might raise some concern because oak forests harbor a particularly high level of biodiversity and might be affected by the spread of Douglas fir.

The success of Douglas fir in resource poor environments might be related to its capability to adjust its root morphology to local conditions. All species increased the fraction of fine root biomass in response to low nutrient availability, but Douglas fir, along with Norway spruce, had the highest fine root fractions when all resources were limited. A high fraction of fine roots allows these two species to more efficiently capture nutrients and water in an environment with highly fluctuating water availability, as for instance in dry regions or on soils with low water retention capacity (Davis et al., 2000). Douglas fir has a higher proportion of fine roots in the topsoil compared to broadleaf species, which generally concentrate their root biomass in a large taproot necessary for the stability of the plants. Although the absence of deep roots in the early life stages may pose a disadvantage during prolonged droughts (Moser et al., 2016), it allows Douglas fir seedlings to efficiently capture nutrients that are concentrated in the organic upper layers of the soil. This likely provides Douglas fir with a distinct advantage under dry and nutrient-poor conditions.

### 4.1 Competitive ability of Douglas fir compared to native conifers

Norway spruce is currently the most important timber species in Switzerland, but it increasingly suffers from bark beetle infestation at low- and mid-elevations, where most commercial timber production occurs (Hlásny et al., 2021). Norway spruce is highly susceptible to destructive disturbances such as winter storms, bark beetle outbreaks, and summer droughts, leading to significant diebacks (e.g., Lévesque et al., 2013, 2014; Obladen et al., 2021; Scherrer et al., 2023). In recent years, the volume of Norway spruce wood from sanitation harvests after dieback events has far exceeded that from scheduled harvests (Wohlgemuth et al., 2023). Consequently, there is a pressing need for an alternative and more drought-resistant coniferous timber species at lower elevations to ensure sustainable wood production under climate warming. Adult Douglas fir is much less affected by bark beetle outbreaks than Norway spruce, ultimately limiting the risk of large dieback (Dubach et al., 2020).

Nevertheless, it is important to thoroughly test the behavior of non-native species and their interactions with extant tree species before promoting them in a new environment (Kreyling et al., 2011; Pedlar et al., 2012; Wohlgemuth et al., 2022). Because Douglas fir is still rare at a national level in Switzerland, it is impossible to draw conclusions about its competitive ability from monitoring data such as national forest inventories. While our common garden findings cannot be directly extrapolated to forest ecosystems, they give important insights in the competitive strength between species and their responsiveness to different environmental factors. Having grown the seedlings from seed in mesocosms avoids legacy effects known to arise when seedlings are raised in nurseries. In our experiment, Douglas fir demonstrated growth and biomass production comparable to, or exceeding, that of Norway spruce, particularly under the anticipated drier conditions of future climates. This was observed even in light conditions corresponding to medium-sized canopy gaps.

Over the first three years, Douglas fir exhibited the most promising growth among the four conifer species studied. It shows potential as a viable alternative to Norway spruce, especially in drier and nutrient-poor sites where it has not yet been widely planted. Being more shade tolerant than Scots pine, and more drought tolerant than Norway spruce, Douglas fir appears best suited to anticipated future conditions on resource limited sites. Due to its high juvenile growth rate, Douglas fir is susceptible to ungulate browsing for a much shorter time than silver fir (Chakraborty et al., 2024), although it is known to be vulnerable to fraying and bark stripping (Nicolescu et al., 2023).

### 4.2 Competitive ability of Douglas fir compared to deciduous broadleaves under future conditions

During the first three years, broadleaf species generally outperformed Douglas fir in both above ground and below ground biomass, as well as in plant height, demonstrating superior competitive ability under both current and future climatic conditions. However, under experimental conditions simulating dryer future conditions, Douglas fir’s 3^rd^ year growth increment surpassed that of all broadleaf species except sycamore. Provided that canopy gaps remain open long enough, Douglas fir thus has the potential to even outgrow many broadleaf species, including climax species like oaks and European beech in Switzerland. Ultimately, the competitive outcome between Douglas fir and fast-growing pioneer and mid-successional species like sycamore in later successional stages will also depend on biotic factors such as ungulate browsing (Petritan et al., 2007; Petrovska et al., 2022).

Even though natural Douglas fir regeneration is currently not abundant in Swiss low and mid-elevation forests (Frei et al., 2021), evidence from our experiment suggests that Douglas fir can successfully regenerate from seeds if propagule pressure is high, i.e. enough seed trees are available in low and mid-elevation forests traditionally dominated by broadleaf species. Its success, however, will depend to a large degree on resource availability, sites conditions (Ammann, 2020), and light transmittance. This aligns with current silvicultural practices in these regions, which have predominantly relied on natural regeneration and are now increasingly combined with planting. While Douglas fir is likely to depend on active support from foresters during the thinning phase to ensure successful establishment and high wood quality on nutrient rich, mesic sites (Frei et al., 2022b), it is important to note that, that Douglas fir has a competitive advantage compared to many species on resource poor sites where it may potentially even become invasive. This is underpinned by observations in oak forests on open, rocky landscape (Knoerzer and Reif, 1996; Höltermann et al., 2013). Accordingly, negative effects of non-native Douglas fir on the biodiversity of various tree- and wood-dependent taxa (for a summary see Wohlgemuth et al., 2021) might be more pronounced in resource poor forests than in nutrient rich sites, where it seems currently not outcompeting native species.

## 5 Conclusion

This study assessed the early growth of Douglas fir compared to native species common in the broadleaf-dominated lowland forests in Central Europe. Our findings revealed that, during the first three years, broadleaf species, including sycamore, generally outperformed Douglas fir and other conifers in terms of biomass and height. However, this early advantage does not necessarily lead to later canopy dominance, as continuous growth is possible only under long-lasting sufficient light conditions. But, Douglas fir demonstrated outstanding growth rates on nutrient-poor soils and under drought conditions in competition with native conifers and even some broadleaves. The adaptability of Douglas fir to dry and poor conditions suggests that it could serve as a viable alternative timber species, particularly in the face of climate change.

Based on our findings, Douglas fir is likely to be able to successfully establish, compete and regenerate in coniferous stands across a large variety of environmental conditions. On sites naturally dominated by broadleaf species, Douglas fir might only be able to successfully recruit on nutrient and water limited sites.

## Data Availability

The datasets presented in this study can be found in the EnviDat repository (https://doi.org/10.16904/envidat.627).
