# Supplementary material for: Non-native Douglas fir seedlings outcompete native Norway spruce, silver fir and Scots pine under dry and nutrient-poor conditions
Source: Front Plant Sci. 2025 Mar 20;16:1546250. doi: 10.3389/fpls.2025.1546250 (PMC11966113; doi:10.3389/fpls.2025.1546250)
Supplement: Supplementary file 1 [file DataSheet1.docx]

**Supplementary figures**


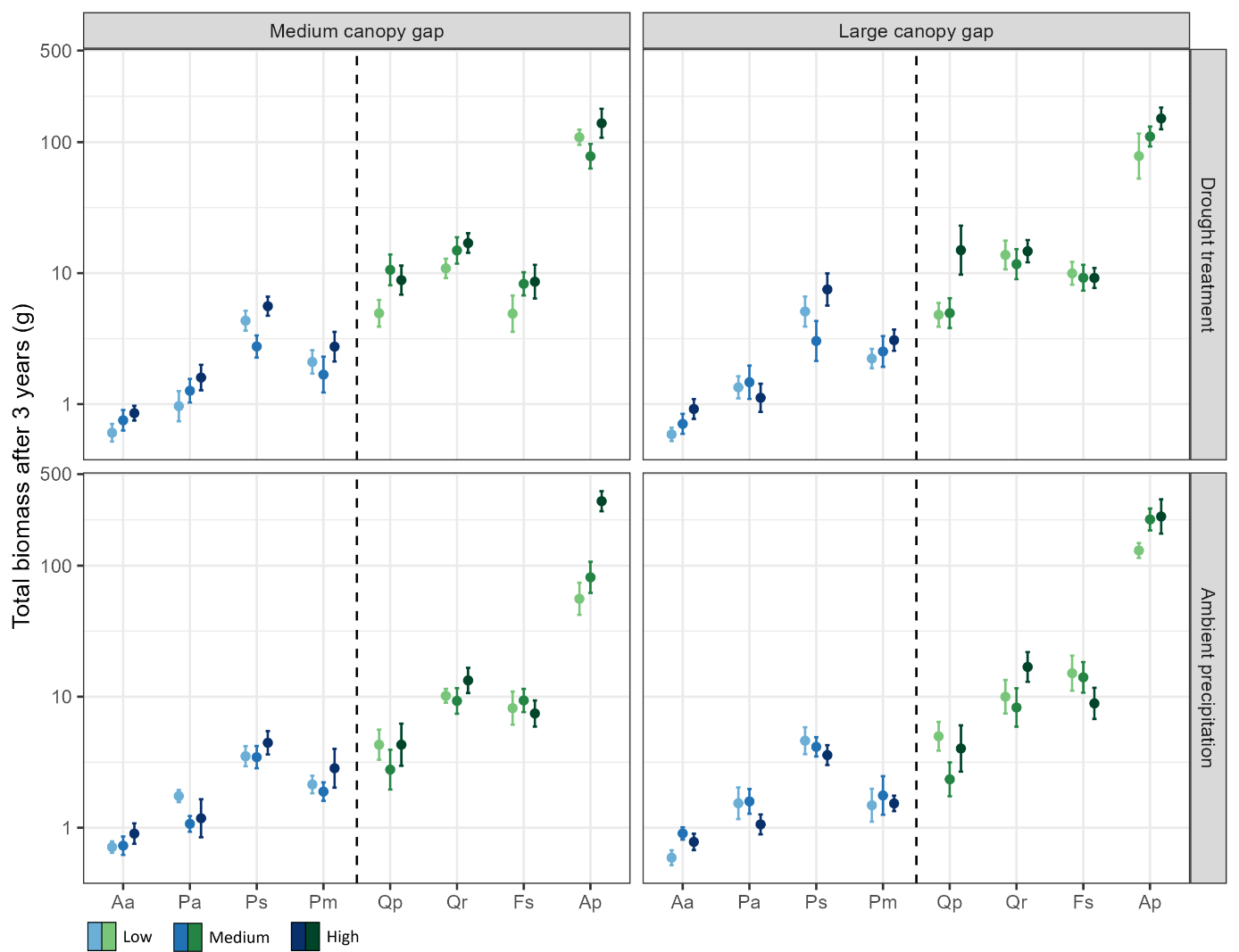


**Figure S1**: Total biomass after three years of the experiment for the eight different plant species across all possible light, water, and nutrient availabilities. Low, medium, and high (light, medium and dark colors) indicate the three different nutrient availabilities. Aa = Silver fir, Pa = Norway spruce, Ps = Scots pine, Pm *=* Douglas fir, Qp = Sessile oak, Qr = Pedunculate oak, Fs = European beech, Ap = Sycamore. Please note that the Y-Axis is displayed as logarithmic scale.

**
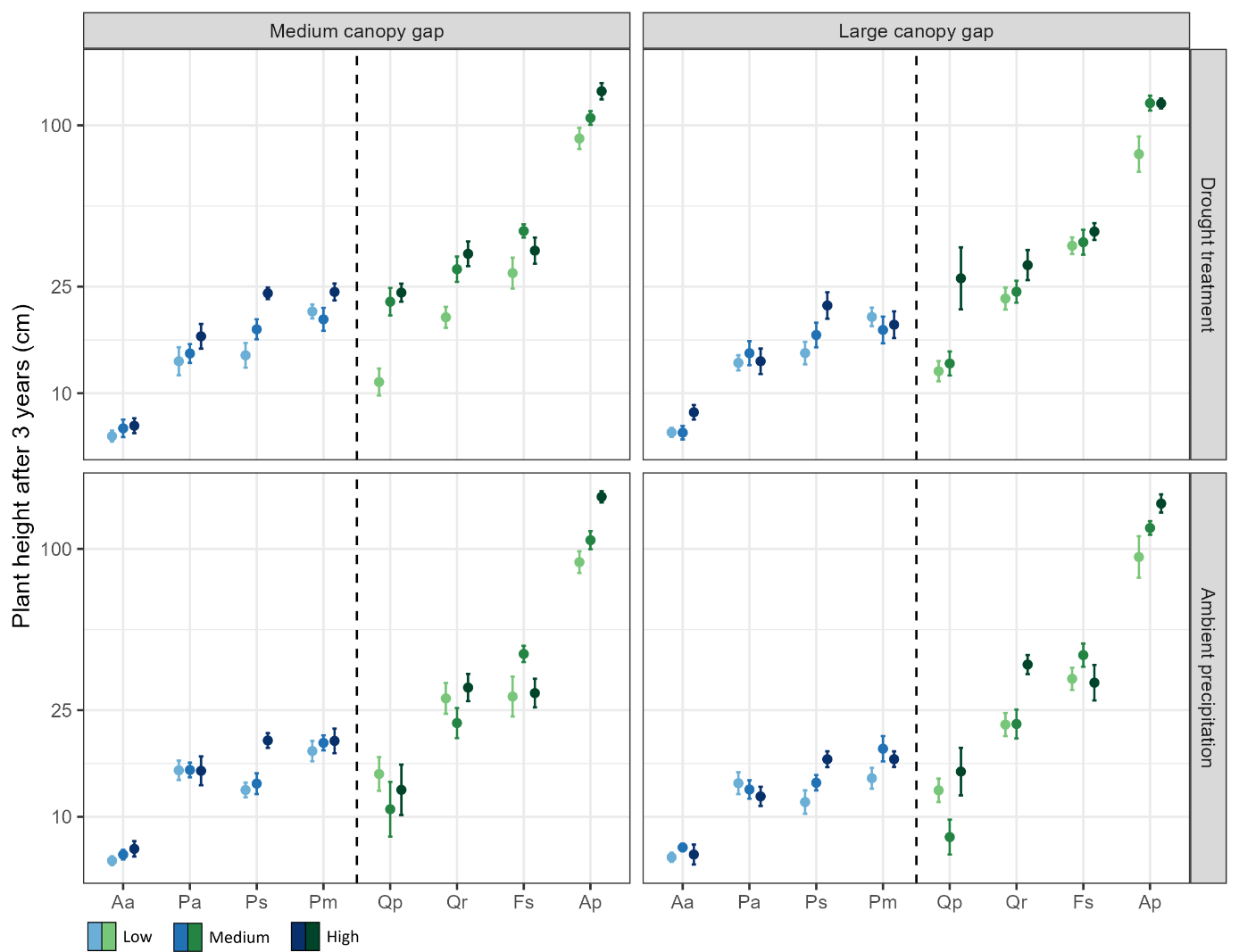
**

**Figure S2**: Plant height after three years of the experiment for the eight different plant species across all possible light, water, and nutrient availabilities. Low, medium, and high (light, medium and dark colors) indicate the three different nutrient availabilities. Aa = Silver fir, Pa = Norway spruce, Ps = Scots pine, Pm *=* Douglas fir, Qp = Sessile oak, Qr = Pedunculate oak, Fs = European beech, Ap = Sycamore. Please note that the Y-Axis is displayed as logarithmic scale.


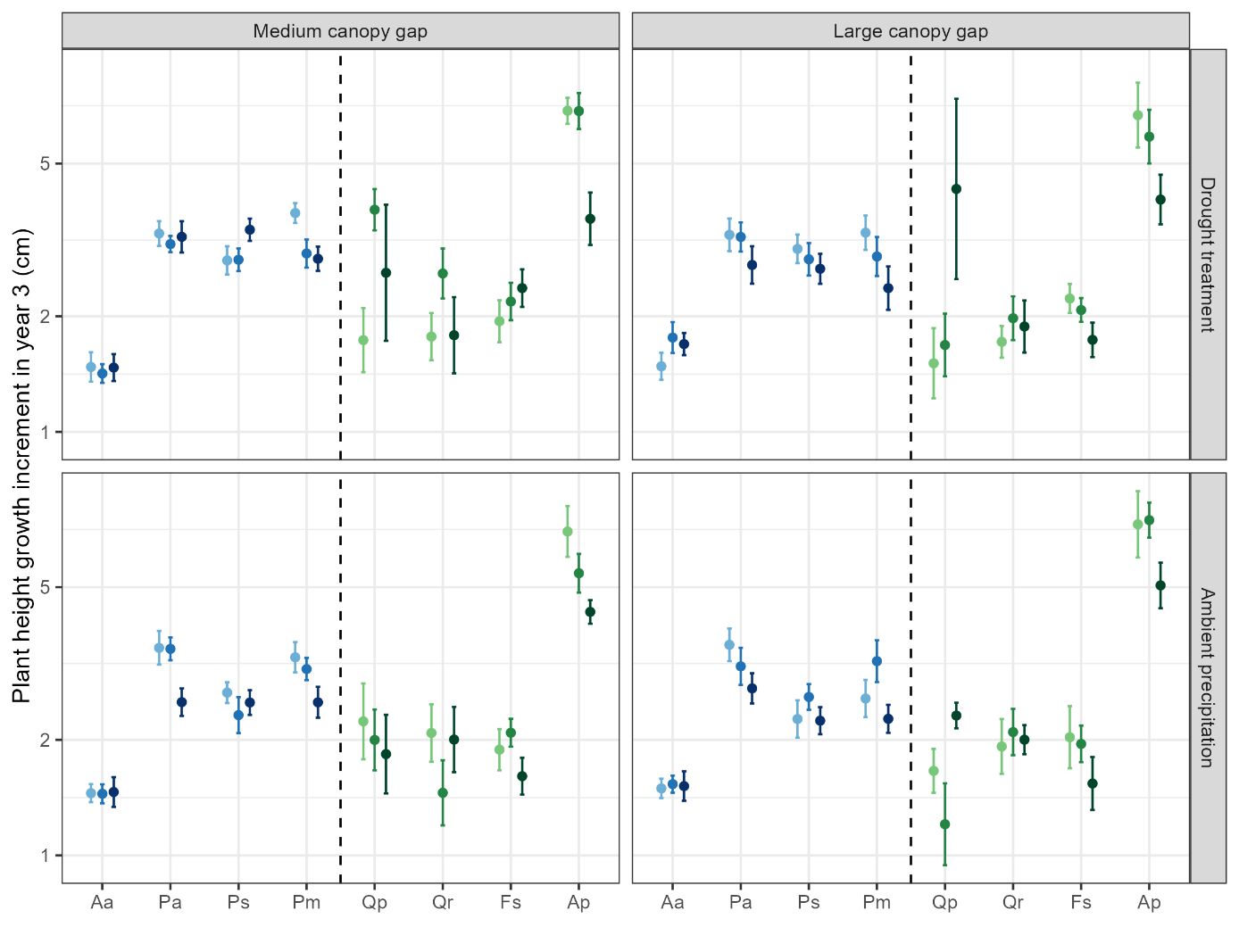


**Figure S3**: Plant height growth increment in the third year of the experiment for the eight different plant species across all possible light, water, and nutrient availabilities. Low, medium, and high (light, medium and dark colors) indicate the three different nutrient availabilities. Aa = Silver fir, Pa = Norway spruce, Ps = Scots pine, Pm *=* Douglas fir, Qp = Sessile oak, Qr = Pedunculate oak, Fs = European beech, Ap = Sycamore. Please note that the Y-Axis is displayed as logarithmic scale.

**
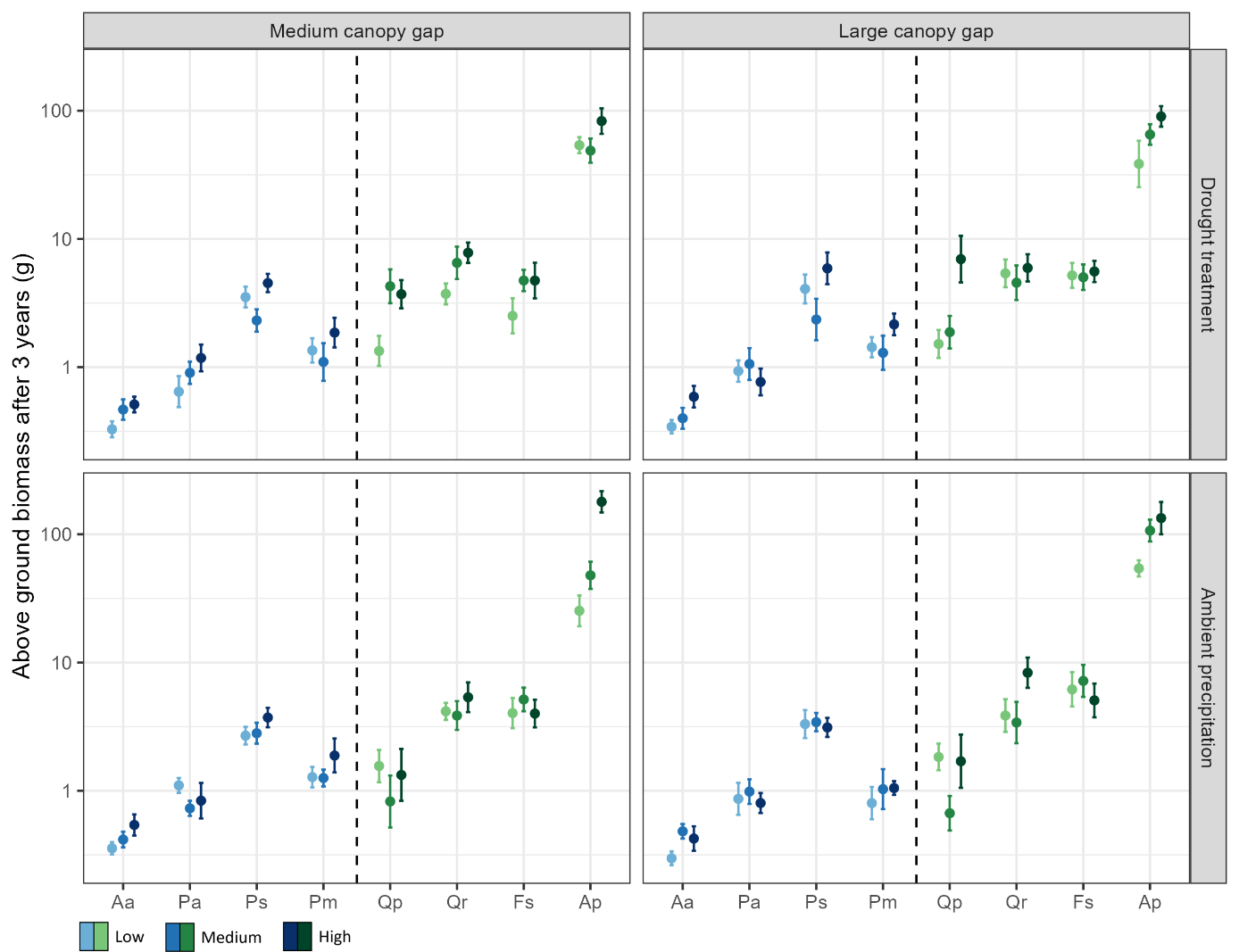
**

**Figure S4**: Above ground biomass after three years of the experiment for the eight different plant species across all possible light, water, and nutrient availabilities. Low, medium, and high (light, medium and dark colors) indicate the three different nutrient availabilities. Aa = Silver fir, Pa = Norway spruce, Ps = Scots pine, Pm *=* Douglas fir, Qp = Sessile oak, Qr = Pedunculate oak, Fs = European beech, Ap = Sycamore. Please note that the Y-Axis is displayed as logarithmic scale.

**
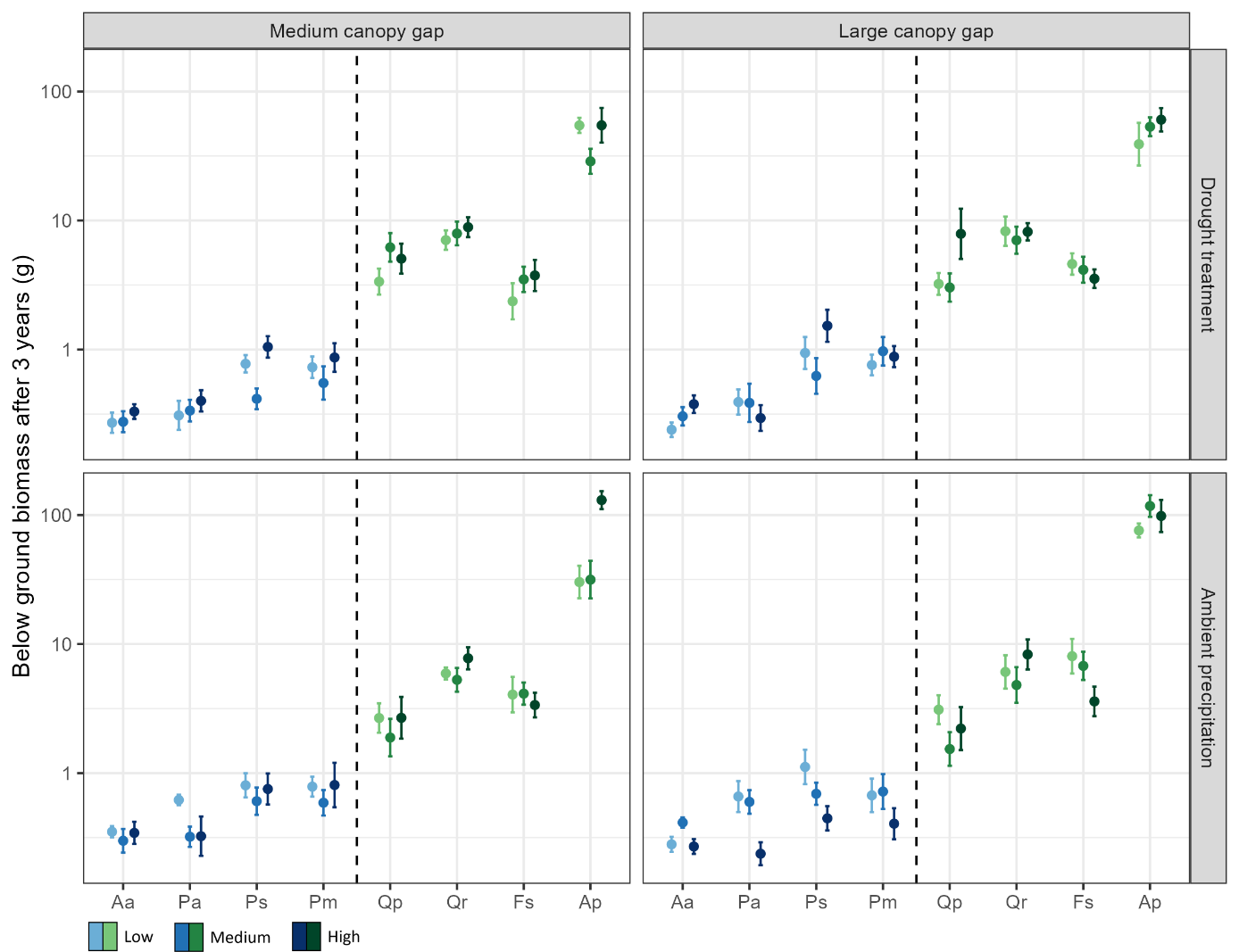
**

**Figure S5**: Below ground biomass after three years of the experiment for the eight different plant species across all possible light, water, and nutrient availabilities. Low, medium, and high (light, medium and dark colors) indicate the three different nutrient availabilities. Aa = Silver fir, Pa = Norway spruce, Ps = Scots pine, Pm *=* Douglas fir, Qp = Sessile oak, Qr = Pedunculate oak, Fs = European beech, Ap = Sycamore. Please note that the Y-Axis is displayed as logarithmic scale.

**
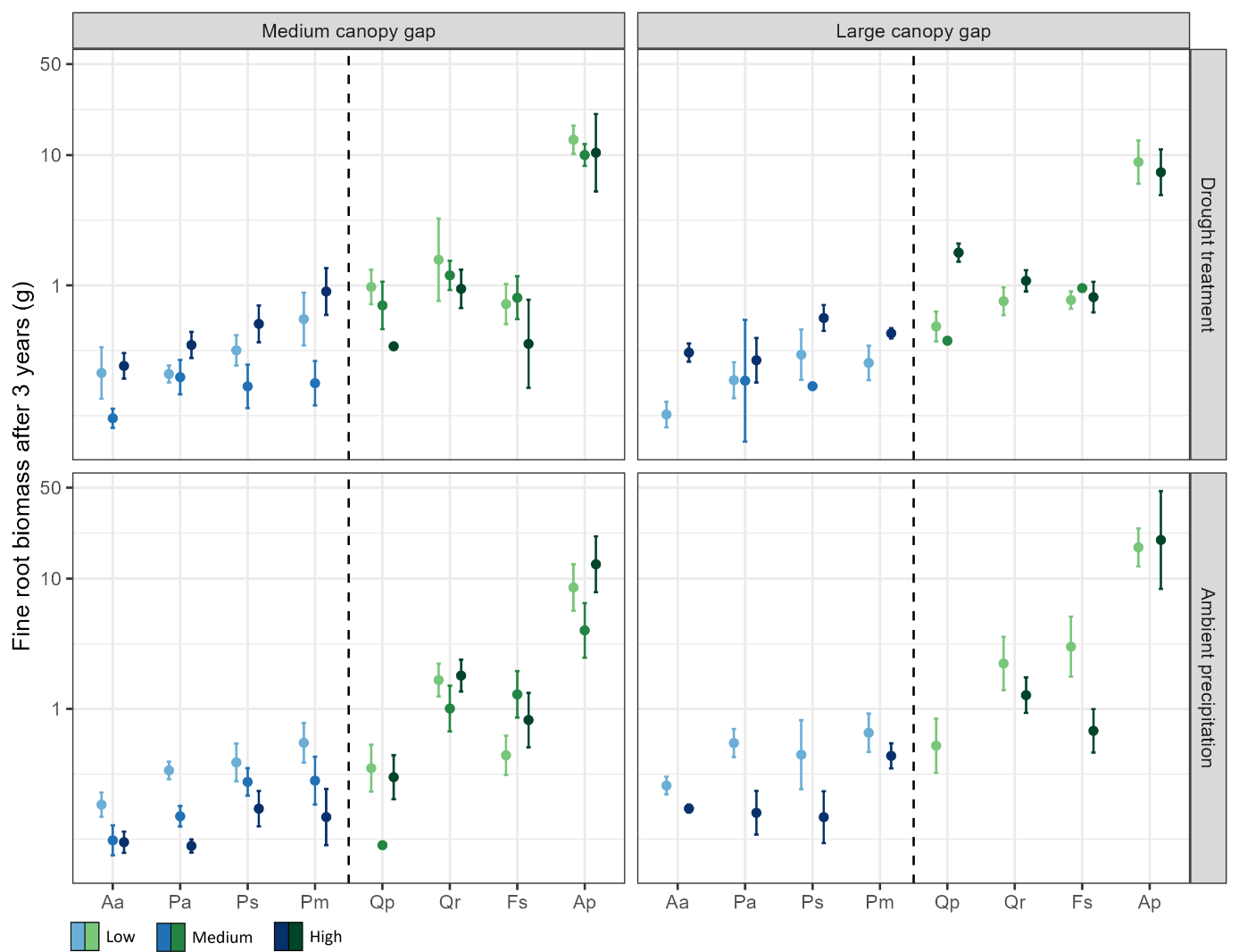
**

**Figure S6**: Fine root biomass after three years of the experiment for the eight different plant species across all possible light, water, and nutrient availabilities. Low, medium, and high (light, medium and dark colors) indicate the three different nutrient availabilities. Aa = Silver fir, Pa = Norway spruce, Ps = Scots pine, Pm *=* Douglas fir, Qp = Sessile oak, Qr = Pedunculate oak, Fs = European beech, Ap = Sycamore. Please note that the Y-Axis is displayed as logarithmic scale.

**
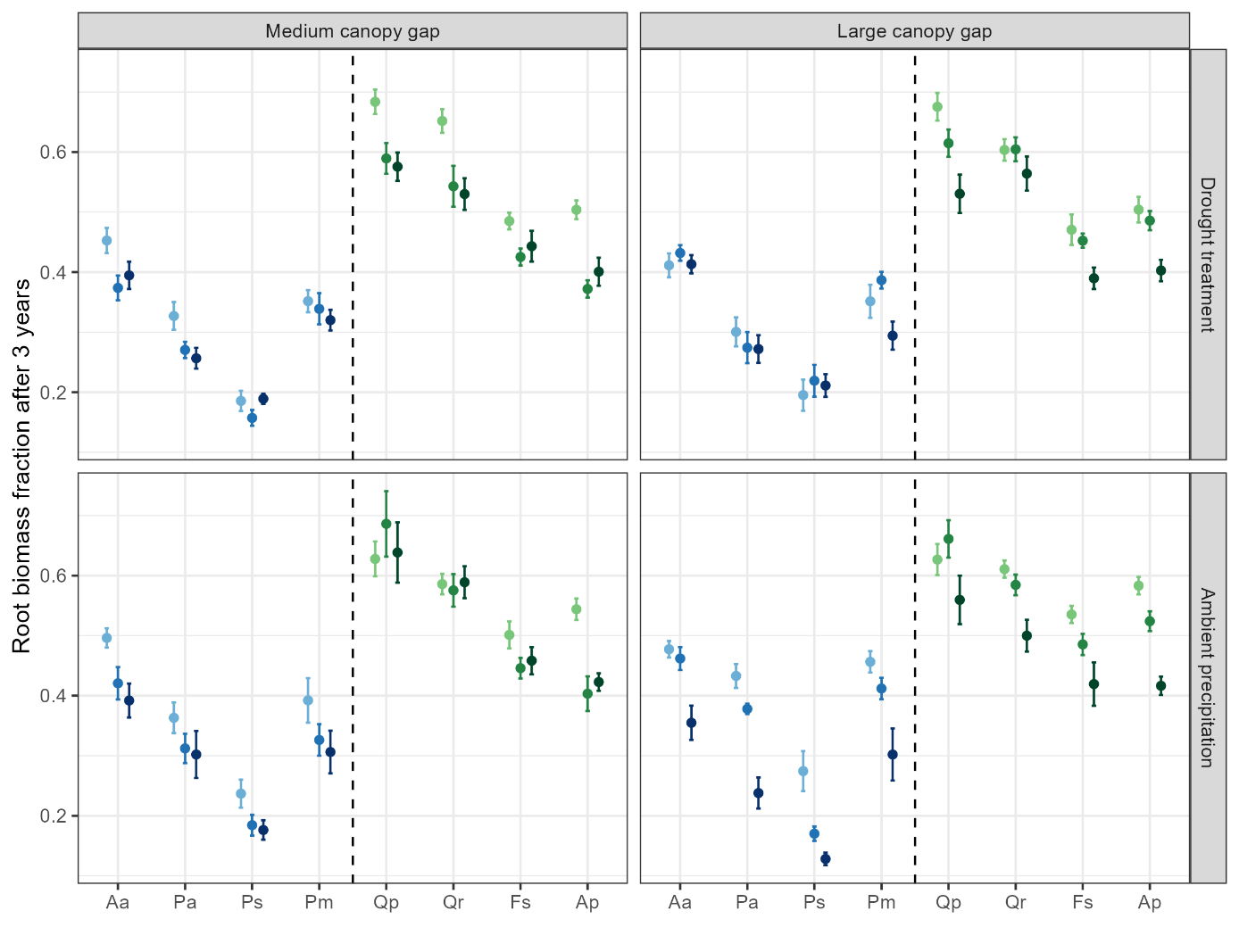
**

**Figure S7**: Root biomass fraction (below ground biomass / total plant biomass) after three years of the experiment for the eight different plant species across all possible light, water, and nutrient availabilities. Low, medium, and high (light, medium and dark colors) indicate the three different nutrient availabilities. Aa = Silver fir, Pa = Norway spruce, Ps = Scots pine, Pm *=* Douglas fir, Qp = Sessile oak, Qr = Pedunculate oak, Fs = European beech, Ap = Sycamore.

**
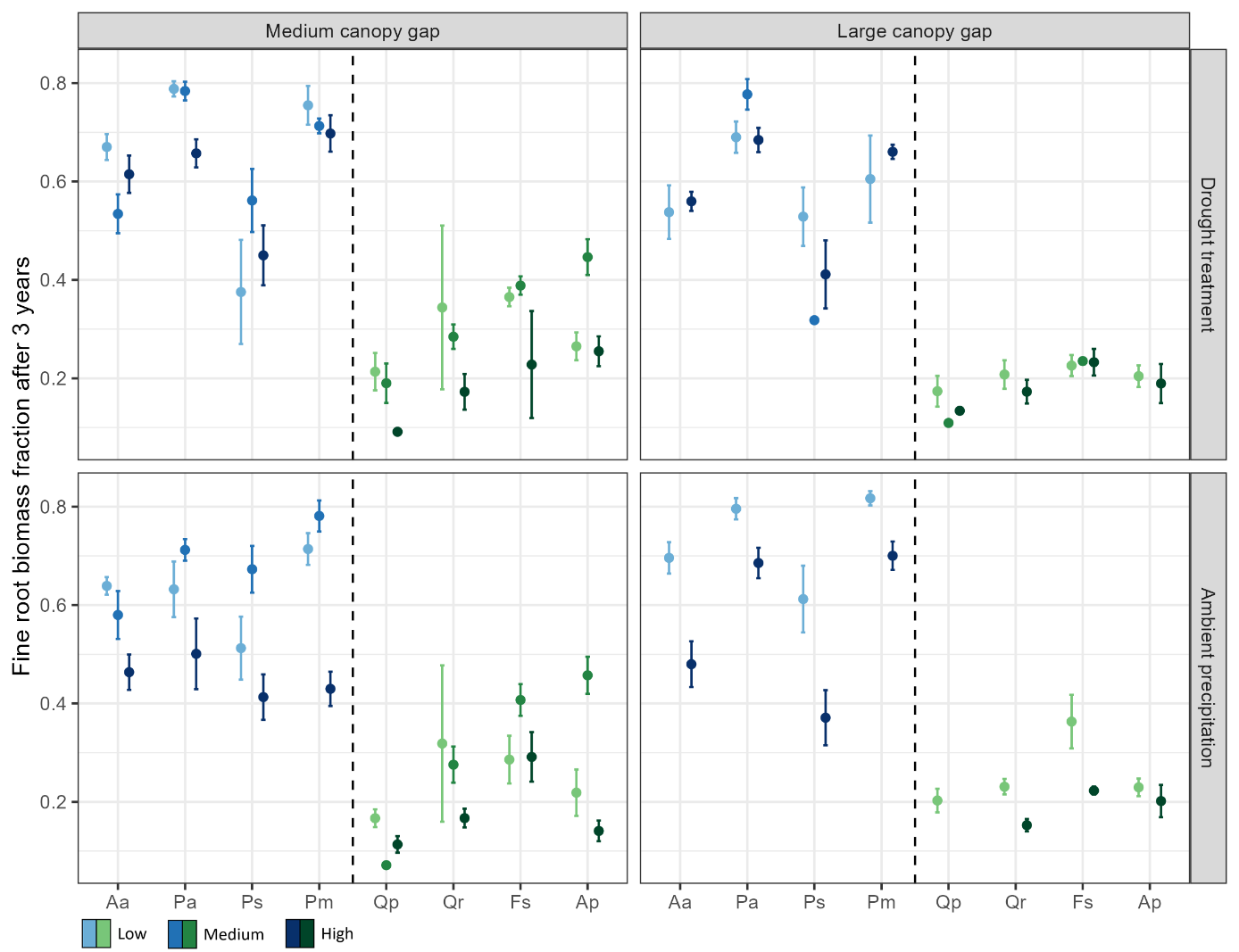
**

**Figure S8**: Fine root fraction (fine root biomass / below ground biomass) after three years of the experiment for the eight different plant species across all possible light, water, and nutrient availabilities. Low, medium, and high (light, medium and dark colors) indicate the three different nutrient availabilities. Aa = Silver fir, Pa = Norway spruce, Ps = Scots pine, Pm *=* Douglas fir, Qp = Sessile oak, Qr = Pedunculate oak, Fs = European beech, Ap = Sycamore.

**
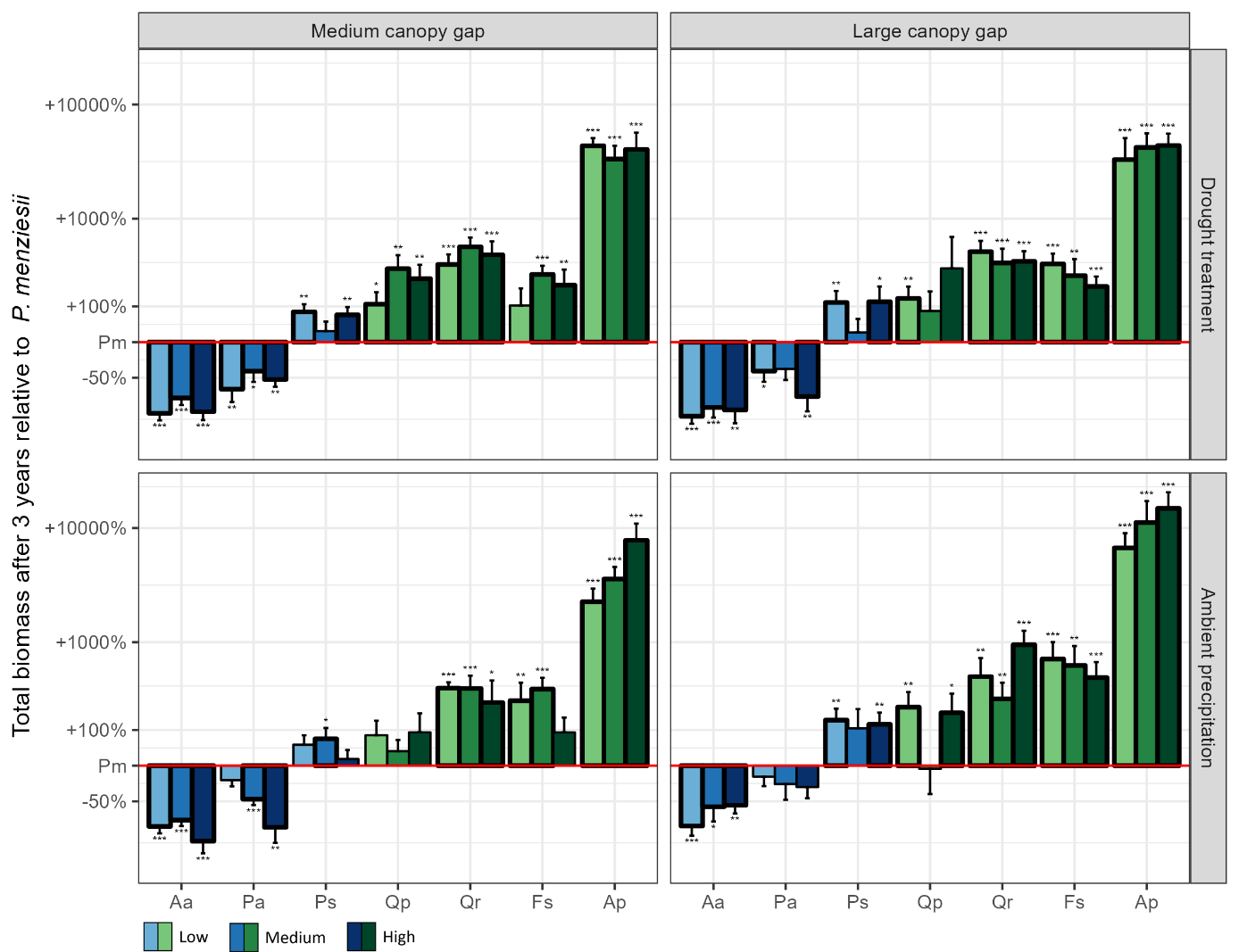
**

**Figure S9**: Total plant biomass after three years of the experiment for the seven native plant species compared to Douglas fir across all possible light, water, and nutrient availabilities. The values of all native plant species were standardized by the corresponding value of Douglas fir in the same treatment combination. Low, medium, and high indicate the three nutrient availabilities. Aa = Silver fir, Pa = Norway spruce, Ps = Scots pine, Pm *=* Douglas fir, Qp = Sessile oak, Qr = Pedunculate oak, Fs = European beech, Ap = Sycamore.

**
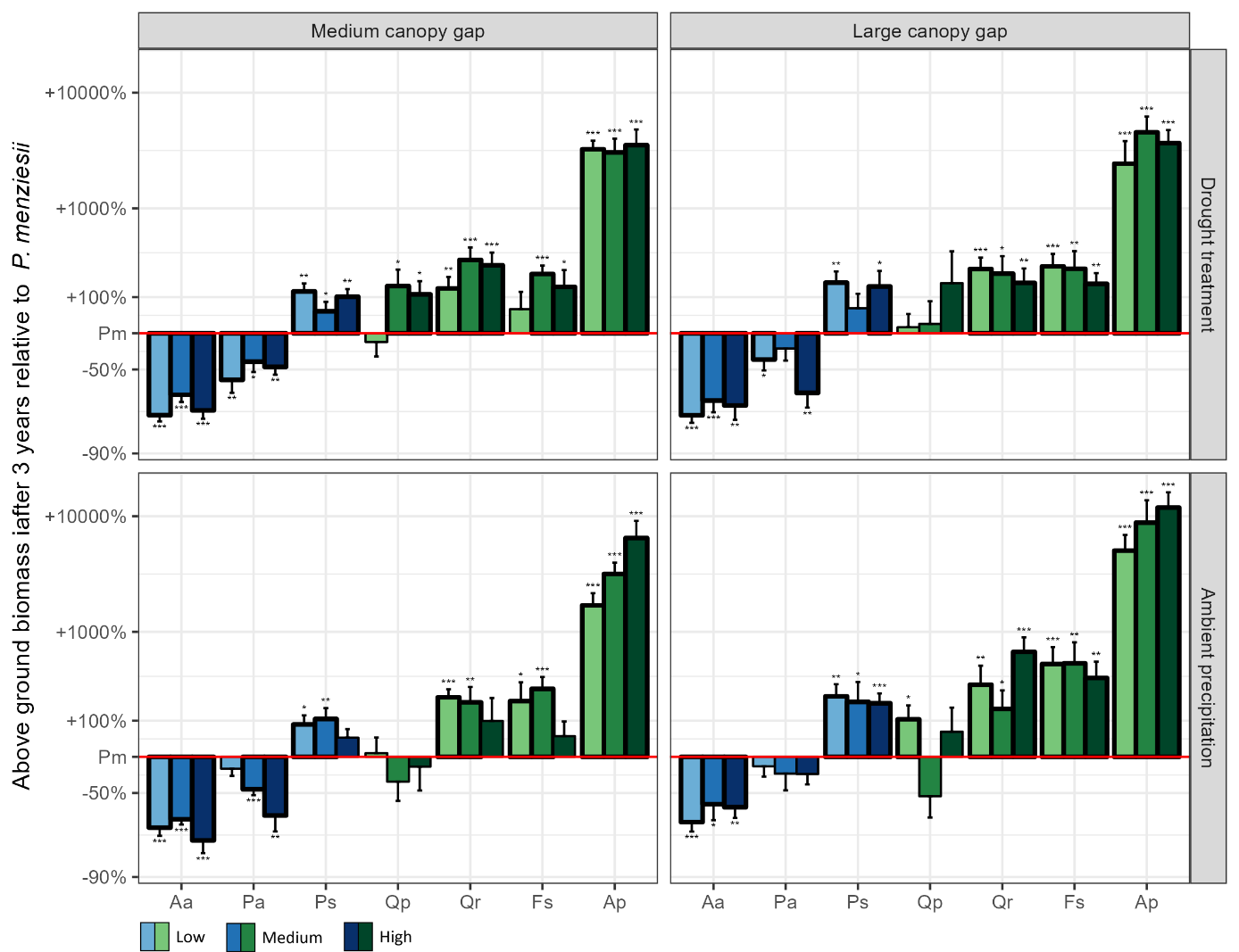
**

**Figure S10**: Above ground biomass after three years of the experiment for the seven native plant species compared to Douglas fir across all possible light, water, and nutrient availabilities. The values of all native plant species were standardized by the corresponding value of Douglas fir in the same treatment combination. Low, medium, and high indicate the three nutrient availabilities. Aa = Silver fir, Pa = Norway spruce, Ps = Scots pine, Pm *=* Douglas fir, Qp = Sessile oak, Qr = Pedunculate oak, Fs = European beech, Ap = Sycamore.


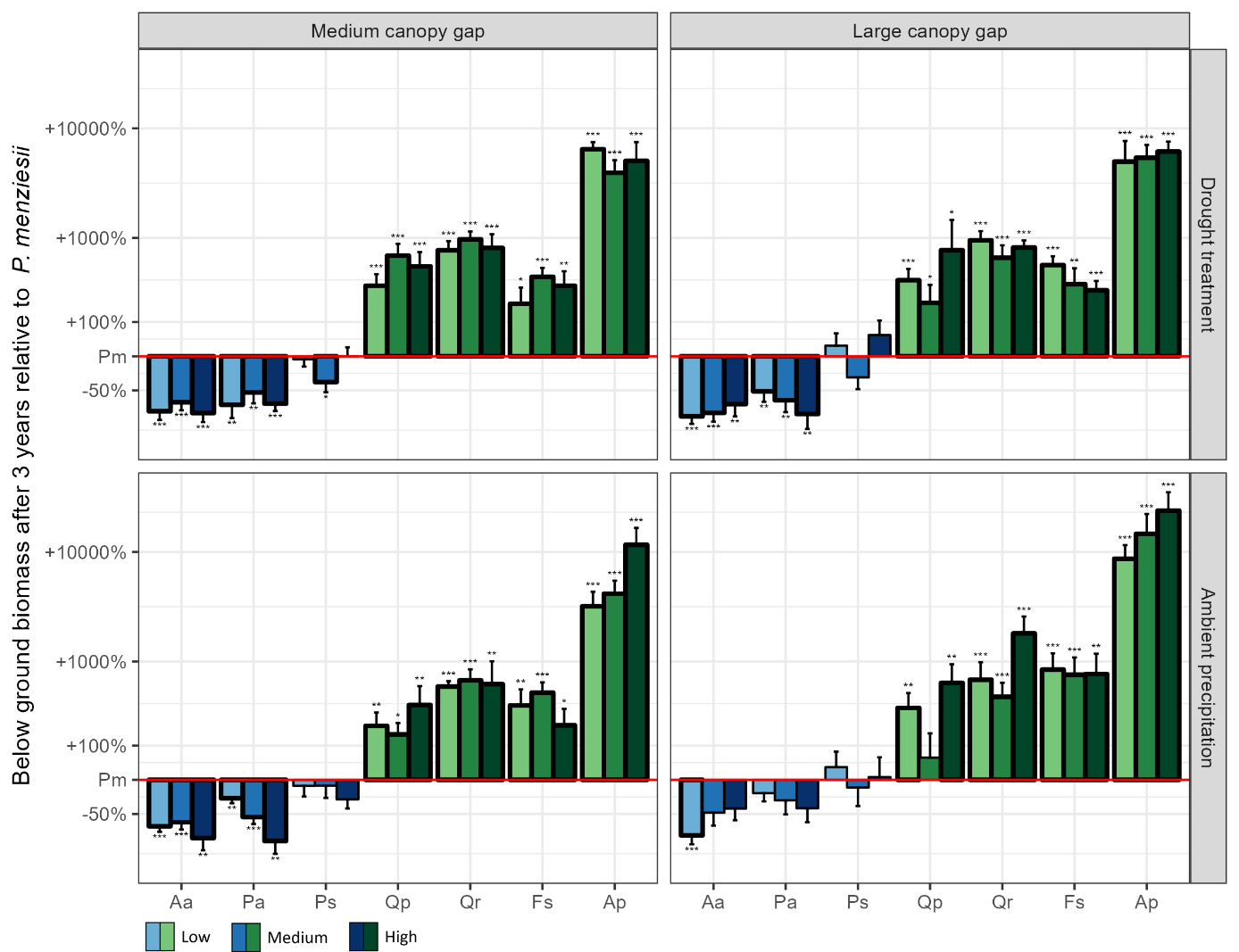


**Figure S11**: Below ground biomass after three years of the experiment for the seven native plant species compared to Douglas fir across all possible light, water, and nutrient availabilities. The values of all native plant species were standardized by the corresponding value of Douglas fir in the same treatment combination. Low, medium, and high indicate the three nutrient availabilities. Aa = Silver fir, Pa = Norway spruce, Ps = Scots pine, Pm *=* Douglas fir, Qp = Sessile oak, Qr = Pedunculate oak, Fs = European beech, Ap = Sycamore.

**
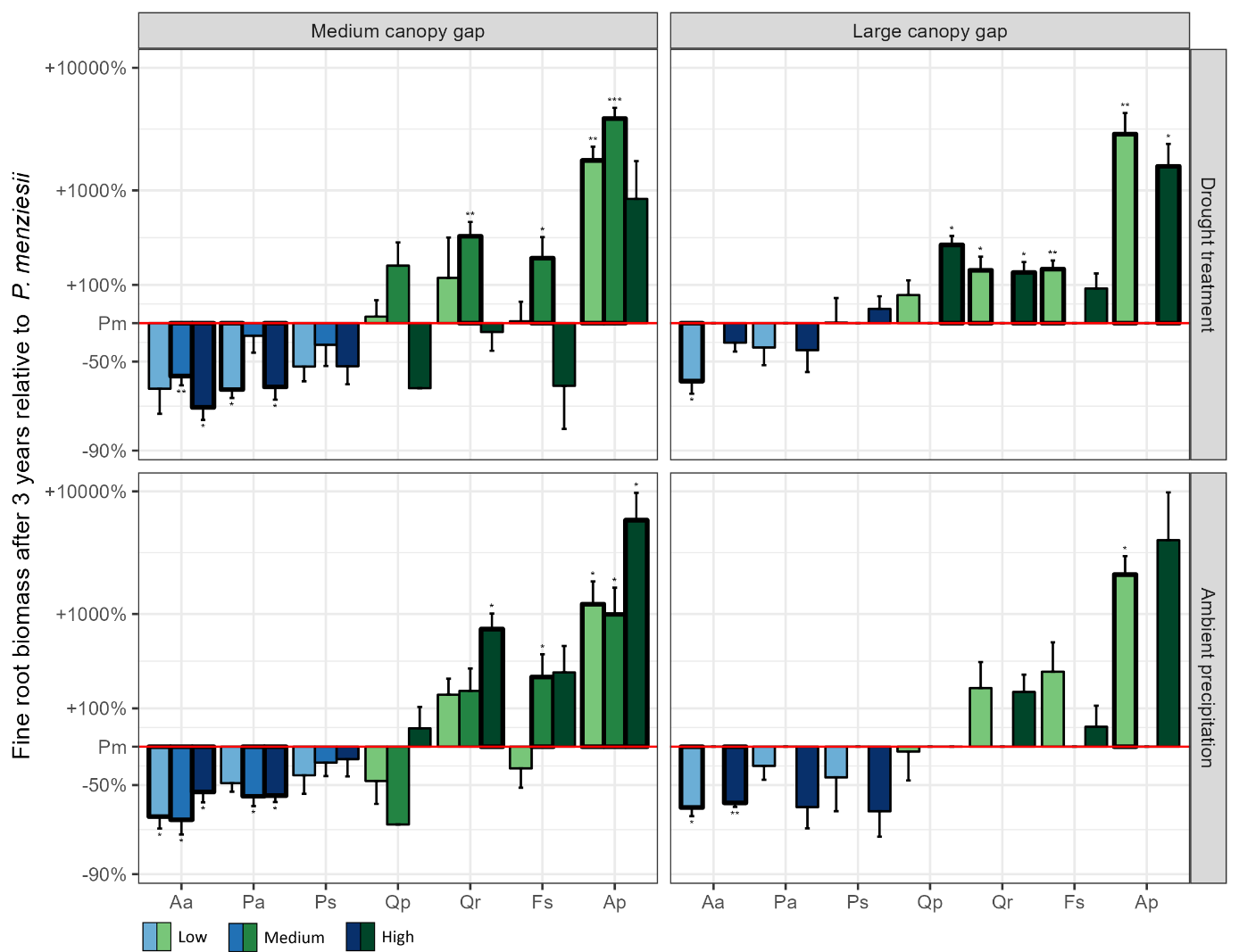
**

**Figure S12**: Fine root biomass after three years of the experiment for the seven native plant species compared to Douglas fir across all possible light, water, and nutrient availabilities. The values of all native plant species were standardized by the corresponding value of Douglas fir in the same treatment combination. Low, medium, and high indicate the three nutrient availabilities. Aa = Silver fir, Pa = Norway spruce, Ps = Scots pine, Pm *=* Douglas fir, Qp = Sessile oak, Qr = Pedunculate oak, Fs = European beech, Ap = Sycamore.

**
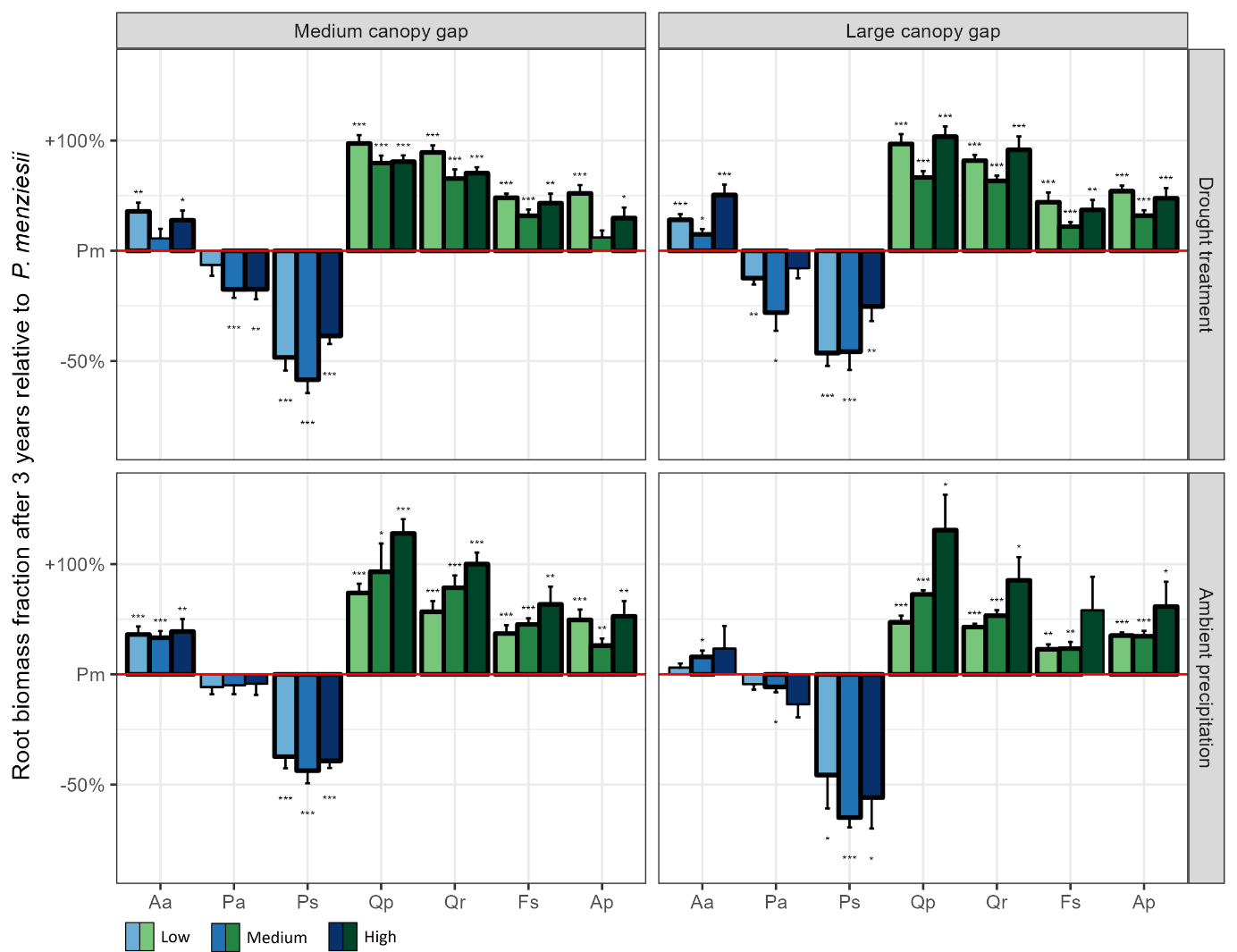
**

**Figure S13**: Root biomass fraction (below ground biomass / total plant biomass) after three years of the experiment for the seven native plant species compared to Douglas fir across all possible light, water, and nutrient availabilities. The values of all native plant species were standardized by the corresponding value of Douglas fir in the same treatment combination. Low, medium, and high indicate the three nutrient availabilities. Aa = Silver fir, Pa = Norway spruce, Ps = Scots pine, Pm *=* Douglas fir, Qp = Sessile oak, Qr = Pedunculate oak, Fs = European beech, Ap = Sycamore.
