# Supplementary material for: Non-native Douglas fir seedlings outcompete native Norway spruce, silver fir and Scots pine under dry and nutrient-poor conditions
Source: Front Plant Sci. 2025 Mar 20;16:1546250. doi: 10.3389/fpls.2025.1546250 (PMC11966113; doi:10.3389/fpls.2025.1546250)
Supplement: Supplementary file 2 [file DataSheet2.docx]

**Table S1**: Seed sources used for the eight different species.

| **Tree species** | | | **Seed source** | **Elevation a.s.l.** |
| --- | --- | --- | --- | --- |
| **Conifers** | | | | |
| Pm | *Pseudotsuga menziesii*  *var. menziesii* | Douglas fir | Washington, USA  (Seed zone 422-20) | 435 |
| Aa | *Abies alba* | Silver fir | Leuk, Switzerland | 1250 |
| Pa | *Picea abies* | Norway spruce | Bremgarten, Switzerland | 425 |
| Ps | *Pinus sylvestris* | Scots pine | Leuk, Switzerland | 630 |
| **Broadleaves** | | | | |
| Ap | *Acer pseudoplatanus* | Sycamore | Arni, Switzerland | 575 |
| Fs | *Fagus sylvatica* | European beech | Hausen a.A., Switzerland | 705 |
| Qr | *Quercus robur* | Pedunculate oak | Aristau, Switzerland | 453 |
| Qp | *Quercus petraea* | Sessile oak | Erlach, BE | 380 |

**Table S2.1**: Linear mixed effects model of the influence of species identity, nutrient availability, water availability and light availability on **plant species height after 3 years**. The experimental setup was accounted for by adding block and mesocosm as nested random factors.

numDF denDF F-value p-value

(Intercept) 1 1387 20503.877 <.0001

**Species 7 1387 960.322 <.0001**

**Fertilizer 2 24 9.906 0.0007**

Precipitation 1 24 1.818 0.1902

Light 1 24 1.165 0.2911

**Species:Fertilizer 14 1387 5.498 <.0001**

**Species:Precipitation 7 1387 4.504 0.0001**

Fertilizer:Precipitation 2 24 0.553 0.5823

Species:Light 7 1387 2.043 0.0468

Fertilizer:Light 2 24 0.195 0.8245

Precipitation:Light 1 24 0.014 0.9071

**Species:Fertilizer:Precipitation 14 1387 2.849 0.0003**

Species:Fertilizer:Light 14 1387 1.337 0.1780

Species:Precipitation:Light 7 1387 0.188 0.9880

Fertilizer:Precipitation:Light 2 24 0.352 0.7065

Species:Fertilizer:Precipitation:Light 14 1387 0.780 0.6922

**Table S2.2**: Linear mixed effects model of the influence of species identity, nutrient availability, water availability and light availability on **plant height growth in the 3^rd^ year**. The experimental setup was accounted for by adding block and mesocosm as nested random factors.

numDF denDF F-value p-value

(Intercept) 1 1179 1965.8905 <.0001

**Species 7 1179 167.9832 <.0001**

**Fertilizer 2 24 4.0941 0.0295**

Precipitation 1 24 1.6784 0.2075

Light 1 24 0.3121 0.5816

**Species:Fertilizer 14 1179 3.7440 <.0001**

**Species:Precipitation 7 1179 1.8617 0.0724**

Fertilizer:Precipitation 2 24 0.1428 0.8677

Species:Light 7 1179 1.7692 0.0897

Fertilizer:Light 2 24 0.0231 0.9772

Precipitation:Light 1 24 0.2016 0.6575

**Species:Fertilizer:Precipitation 14 1179 1.7252 0.0454**

Species:Fertilizer:Light 14 1179 1.3356 0.1789

Species:Precipitation:Light 7 1179 0.5653 0.7845

Fertilizer:Precipitation:Light 2 24 0.3955 0.6776

Species:Fertilizer:Precipitation:Light 14 1179 0.8280 0.6388

**Table S2.3**: Linear mixed effects model of the influence of species identity, nutrient availability, water availability and light availability on **total plant biomass after 3 years**. The experimental setup was accounted for by adding block and mesocosm as nested random factors.

numDF denDF F-value p-value

(Intercept) 1 866 1306.3301 <.0001

**Species 7 866 616.9462 <.0001**

Fertilizer 2 24 2.5135 0.1021

Precipitation 1 24 0.4200 0.5231

Light 1 24 0.3878 0.5393

**Species:Fertilizer 14 866 2.2360 0.0056**

**Species:Precipitation 7 866 4.2730 0.0001**

Fertilizer:Precipitation 2 24 0.3150 0.7328

Species:Light 7 866 1.6804 0.1103

Fertilizer:Light 2 24 0.4877 0.6200

Precipitation:Light 1 24 0.0012 0.9725

**Species:Fertilizer:Precipitation 14 866 1.8633 0.0268**

Species:Fertilizer:Light 14 866 1.0402 0.4102

Species:Precipitation:Light 7 866 1.2516 0.2717

Fertilizer:Precipitation:Light 2 24 0.3605 0.7010

Species:Fertilizer:Precipitation:Light 14 866 0.7680 0.7048

**Table S2.4**: Linear mixed effects model of the influence of species identity, nutrient availability, water availability and light availability on **above ground biomass after 3 years**. The experimental setup was accounted for by adding block and mesocosm as nested random factors.

numDF denDF F-value p-value

(Intercept) 1 884 538.3962 <.0001

**Species 7 884 521.3236 <.0001**

**Fertilizer 2 24 5.4640 0.0111**

Precipitation 1 24 1.2638 0.2720

Light 1 24 0.1596 0.6931

**Species:Fertilizer 14 884 2.4439 0.0022**

**Species:Precipitation 7 884 3.2499 0.0021**

Fertilizer:Precipitation 2 24 0.2713 0.7647

Species:Light 7 884 1.3517 0.2227

Fertilizer:Light 2 24 0.3683 0.6957

Precipitation:Light 1 24 0.0040 0.9502

**Species:Fertilizer:Precipitation 14 884 2.5560 0.0013**

Species:Fertilizer:Light 14 884 0.9691 0.4833

Species:Precipitation:Light 7 884 0.9813 0.4434

Fertilizer:Precipitation:Light 2 24 0.3185 0.7302

Species:Fertilizer:Precipitation:Light 14 884 0.8975 0.5614

**Table S2.5**: Linear mixed effects model of the influence of species identity, nutrient availability, water availability and light availability on **below ground biomass after 3 years**. The experimental setup was accounted for by adding block and mesocosm as nested random factors.

numDF denDF F-value p-value

(Intercept) 1 867 198.7863 <.0001

**Species 7 867 730.9763 <.0001**

Fertilizer 2 24 0.2940 0.7479

Precipitation 1 24 0.0283 0.8677

Light 1 24 0.5651 0.4595

**Species:Fertilizer 14 867 2.5625 0.0013**

**Species:Precipitation 7 867 5.1061 <.0001**

Fertilizer:Precipitation 2 24 0.7553 0.4807

Species:Light 7 867 1.8522 0.0744

Fertilizer:Light 2 24 1.0499 0.3655

Precipitation:Light 1 24 0.0531 0.8197

**Species:Fertilizer:Precipitation 14 867 1.9658 0.0177**

Species:Fertilizer:Light 14 867 1.3385 0.1780

Species:Precipitation:Light 7 867 1.3089 0.2427

Fertilizer:Precipitation:Light 2 24 0.5606 0.5781

Species:Fertilizer:Precipitation:Light 14 867 0.6473 0.8260

**Table S2.6**: Linear mixed effects model of the influence of species identity, nutrient availability, water availability and light availability on **the fraction of below-ground biomass**. The experimental setup was accounted for by adding block and mesocosm as nested random factors.

numDF denDF F-value p-value

(Intercept) 1 888 2131.5348 <.0001

**Species 7 888 429.9540 <.0001**

**Fertilizer 2 888 91.0007 <.0001**

**Precipitation 1 888 21.0652 <.0001**

**Light 1 888 8.0008 0.0048**

Species:Fertilizer 14 888 1.5633 0.0836

**Species:Precipitation 7 888 2.2238 0.0304**

**Fertilizer:Precipitation 2 888 5.5891 0.0039**

Species:Light 7 888 2.0165 0.0504

**Fertilizer:Light 2 888 20.3178 <.0001**

Precipitation:Light 1 888 0.0201 0.8872

**Species:Fertilizer:Precipitation 14 888 2.7557 0.0005**

Species:Fertilizer:Light 14 888 0.7993 0.6707

Species:Precipitation:Light 7 888 1.4440 0.1841

**Fertilizer:Precipitation:Light 2 888 8.2074 0.0003**

Species:Fertilizer:Precipitation:Light 14 888 0.6356 0.8366
